# Efficacy of Internet-Based Cognitive Behavioral Therapy for Subthreshold Depression Among Older Adults in Institutional Long-Term Care Settings: Pragmatic Randomized Controlled Trial

**DOI:** 10.2196/40187

**Published:** 2024-03-01

**Authors:** Fanqian Kong, Libo Yu, Yanbin Hou, Lijie Zhu, Jing Zhou, Lingjie Huang, Yueer Lv, Li Wang, Li Zhang, Yiling Yang, Yuchen Ying

**Affiliations:** 1 Ningbo Medical Center Lihuili Hospital Ningbo China; 2 Ningbo College of Health Sciences Ningbo, Zhejiang China; 3 Ningbo First Hospital Ningbo China; 4 Pfizer Research and Development Co, Ltd Shanghai China

**Keywords:** internet-based cognitive behavioral therapy, subthreshold depression, randomized controlled trial, older adults, LTC setting

## Abstract

**Background:**

Subthreshold depression (sD) is prevalent in older populations in long-term care (LTC) settings, but psychological therapy in LTC settings in China is not readily available. Thus, internet-based cognitive behavioral therapy (ICBT) may be suitable for this population, but research on the efficacy of ICBT for older adults with sD, especially those living in LTC settings, is limited.

**Objective:**

This study aimed to evaluate the efficacy and acceptability of ICBT treatment for sD among LTC residents in China. We also examined whether ICBT is as effective as group-based cognitive behavioral therapy (CBT) for treating sD in this population.

**Methods:**

We conducted a pragmatic randomized controlled trial, which included 18 LTC institutions. A total of 354 participants were randomized to ICBT, group-based CBT, or a waiting list and were followed up for 12 months. The primary outcome was self-reported depressive symptoms on the Center for Epidemiological Studies Depression Scale (CES-D). Secondary outcomes were the scores of the Patient Health Questionnaire–9 (PHQ-9), Generalized Anxiety Disorder 7-Item (GAD-7), and Geriatric Depression Scale (GDS). A mixed-effects model was used to assess the efficacy of ICBT.

**Results:**

The ICBT group showed a significant improvement in self-reported depressive symptoms, which was maintained at the 12-month follow-up (all *P*<.001). The ICBT group exhibited a significantly larger reduction in the scores of the CES-D (Cohen *d*=0.07, 95% CI 0.04-0.09; *P*=.01), PHQ-9 (*d*=0.30, 95% CI 0.28-0.33; *P*<.001), GDS (*d*=0.10, 95% CI 0.08-0.13; *P*<.001), and GAD-7 (*d*=0.19, 95% CI 0.17-0.22; *P*<.001) compared with a waiting list at postintervention. ICBT had significantly stronger effects than CBT on the PHQ-9 and GAD-7 at postintervention (*d*=0.15, 95% CI 0.13-0.17; *P*<.001 and *d*=0.21, 95% CI 0.19-0.23; *P*<.001, respectively), 6-month follow-up (*d*=0.18, 95% CI 0.16-0.21; *P*<.001 and *d*=0.18, 95% CI 0.15-0.21; *P*<.001, respectively), and 12-month follow-up (*d*=0.15, 95% CI 0.11-0.19; *P*<.001 and *d*=0.18, 95% CI 0.14-0.21; *P*<.001, respectively).

**Conclusions:**

ICBT is a relatively effective and acceptable intervention for reducing depressive symptoms among Chinese LTC residents with sD. These findings indicate the usefulness of ICBT application for sD in LTC settings.

**Trial Registration:**

Chinese Clinical Trial Registry ChiCTR2000030697; https://www.chictr.org.cn/showproj.aspx?proj=50781

## Introduction

Subthreshold depression (sD) is a significant health burden for older adults [1]. It is even more prevalent than diagnosed clinical depression among older adults [2] and adversely affects their social functions [3], activities of daily living [4], and quality of life [5]. In addition, it is related to increased risk of major depression [6], cognitive impairment and dementia [7], physical ailments [8], and increased mortality and medical costs in older adults [9]. Furthermore, epidemiological research on older adults with sD has revealed a pattern: increasing rates of moving from community to primary care to long-term care (LTC) settings [10]. Therefore, it is necessary to develop appropriate interventions to treat sD among older adults, especially those living in LTC settings.

With respect to the treatment of depression, older adults prefer psychotherapy to pharmacotherapy [11]; cognitive behavioral therapy (CBT) has proven effective [12]. However, the treatment rate for older adults is low due to numerous barriers to face-to-face CBT, including mobility limitations, relatively high medical costs, limited number of trained therapists, and stigma [13]. Fortunately, internet-based CBT (ICBT), an intervention that can be conducted anytime and anywhere over the internet, can overcome these barriers [14]. Nevertheless, although several studies have demonstrated that ICBT may significantly reduce depressive symptoms in older adults with moderate to severe depression [15], the impact of ICBT on older adults with sD is unclear. In addition, although preliminary evidence demonstrated that guided ICBT and face-to-face CBT might be equally effective [16], only 1 prior study has demonstrated that ICBT is more effective than face-to-face CBT in treating sD among older adults living in the community [17]. However, this study did not provide a between-group effect size on the outcomes of ICBT versus CBT, and, therefore, whether ICBT is as effective as face-to-face CBT among LTC residents with sD remains unknown.

Many older Chinese adults are unlikely to seek face-to-face psychotherapy due to stigma that discourages disclosure influenced by Chinese cultural values; thus, undertreatment is common among older adults in China [18]. Of note, the overall detection rate of mild depressive symptoms among Chinese LTC residents is only 30% [19]. Considering China’s 2.15 million LTC residents and the extreme shortage of its mental health workforce, especially psychological therapists in LTC settings [20], many cases of sD might go undetected and untreated. To address this issue, the Chinese government released an action plan for depression prevention and treatment to improve access to web interventions (eg, ICBT) for depression among older adults [21]. However, the efficacy and acceptability of ICBT among the older adult Chinese population, especially those in LTC settings, have not been investigated. Therefore, evidence-based research is needed to promote the use of ICBT in LTC settings in China. LTC settings in China are broadly divided into two categories: (1) institutional settings and (2) home- and community-based settings [22]. This study focused on institutional LTC facilities because they have a somewhat similar definition to that of LTC settings in previous studies [10].

The purpose of this study was to test the following hypotheses: (1) ICBT is more effective than a waiting list (WL) in improving depressive symptoms and other secondary outcomes, (2) ICBT is equally effective as group CBT in improving depressive symptoms and other secondary outcomes, and (3) ICBT is effective and acceptable for Chinese LTC residents.

## Methods

### Study Design

This study was a pragmatic, multicenter randomized controlled trial (RCT) with 3 arms comparing clinician-guided ICBT with group CBT and a WL control. The patients were assessed at baseline, immediately after the intervention, and at 6- and 12-month follow-ups.

The interventions and WL control were not compared at 6- and 12-month follow-ups because the WL group had only 2 assessment points (ie, baseline and postintervention).

### Ethical Considerations 

This study was approved by the Medical Ethics Committee of Ningbo University (approval number: NBU-2020-139) and registered in the Chinese Clinical Trials Registry (registration ChiCTR2000030697).

### Participants and Procedures

A total of 618 participants were recruited via 18 LTC settings across 6 cities in Zhejiang Province. The choice of the cities and LTC settings ensured that the sample included participants from locations with different population compositions.

To protect the residents’ privacy, initial contact was made by the designated research liaison staff member at each LTC setting. This person asked about the residents’ willingness to participate in the study, examined their health records, and identified eligible participants based on our screening criteria. The inclusion criteria were as follows: (1) be a resident of China, (2) be 60 years or older, (3) have access to the internet, (4) have sD (defined as having a score of ≥16 on the Centre for Epidemiological Studies Depression Scale [CES-D], according to the widely used criteria in comparable studies [6,23]), (5) no suicidal ideation (indicated by a score of <2 on question 9 on the Patient Health Questionnaire–9 [PHQ-9]) [24], (6) no cognitive impairment with a score on a Mini-Mental State Examination >24 [25], (7) not currently undergoing psychotherapy, (8) no alcohol dependency, and (9) no terminal illness on clinical grounds.

**Figure 1:**
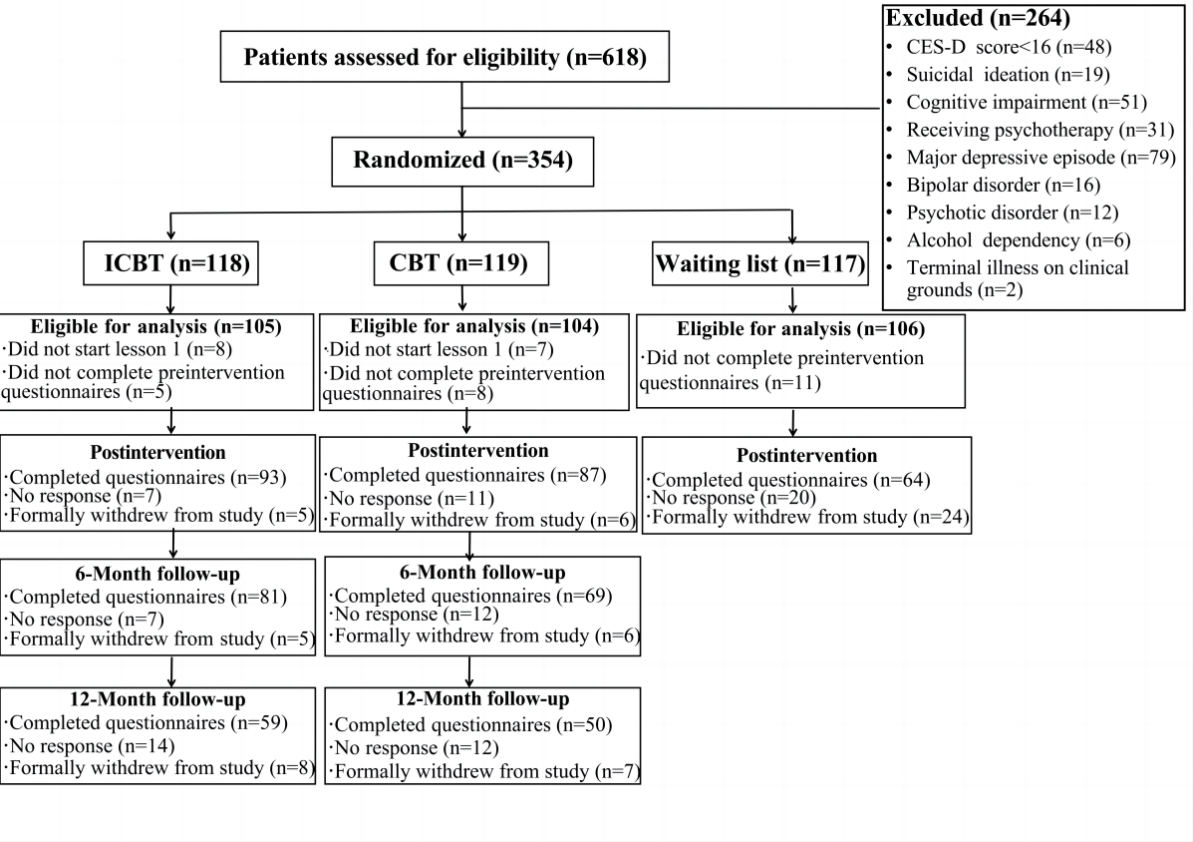
Flowchart of participants. CBT: cognitive behavioral therapy; CES-D: Center for Epidemiological Studies Depression Scale; ICBT: internet-based cognitive behavioral therapy.

The staff invited potential eligible participants to join the study after obtaining written informed consent. These potential participants were further invited for the telephone-administered Semistructured Clinical Interview for Diagnostic and Statistical Manual of Mental Disorders, Fourth Edition (DSM-IV) Axis Disorders (SCID) to assess final eligibility. The exclusion criteria were as follows: meeting the DSM-IV criteria for (1) major depressive disorder (MDD), (2) bipolar disorder, or (3) psychotic disorder. Participants on antidepressants remained eligible if they were on a stable dose for the past 4 weeks and had no change in medication during the study. New residents who met the inclusion criteria could also be invited. Standardized questionnaires including all measures and sociodemographic data were administered to eligible participants. Of the recruited participants, 354 met the inclusion criteria. The participants were stratified by age, sex, study site, use of antidepressants, and depression symptom severity (determined using CES-D scores) and then randomized to ICBT (n=118), CBT (n=119), or WL (n=117). Figure 1 depicts the flow of participants through the trial.

Randomization was performed at a ratio of 1:1:1 in blocks of 6 using a web randomizer by an external researcher from another institution. Each allocation was kept in sequentially numbered, sealed opaque envelopes. Clinical psychologists conducting SCID were blinded to the group assignments. Except for the WL group as a control, none of the participants were blinded to group allocation. Participants who completed postintervention and follow-up questionnaires received CN ¥75 (approximately US $11.80).

### Interventions

#### Clinician-Guided ICBT

This clinician-guided ICBT is based on the principle of the Chinese version of the *Handbook of Cognitive-Behavioral Therapies* [26]. In this trial, the depression module was used. This module comprises 5 lessons with corresponding case stories, which were developed from real cases of Chinese patients who recovered from sD or MDD after receiving CBT, and was designed to educate the general Chinese population about practical CBT skills for depression [27]. Textbox 1 depicts each lesson’s descriptions. Although the duration of lessons could vary among participants, they were advised to undertake at least 1 lesson per week in an individual format. The role of clinicians, who were blinded to the hypotheses, was to provide technical assistance and encouragement to participants during the study.

**Lesson content of the depression module.**

**Lesson 1**

- Lesson content

Getting support, building relationship, and signing contracts

Introduction to listening, empathy, active attention, depression, cognitive behavioral therapy, psychological education, etc

Understanding, relationship building, and cognitive assessment (preliminary establishment of case conceptualization and treatment plan)

- Case story
  Instructions and examples of building a relationship and signing contracts with clinicians
  Instructions and examples of assessing own symptoms of depression
  Instructions and examples of establishing cognitive case conceptualization and treatment plan

**Lesson 2**

- Lesson content
  Introduction to the psychological meaning of symptoms of depression
  Introduction to the role of cognition (cognitive recognition, behavioral function analysis, cognitive behavior recording, reality test, etc)
- Case story
  Instructions and examples of cognitive recognition, behavioral function analysis, cognitive behavior recording, and reality test

**Lesson 3**

- Lesson content
  Introduction to the relationship between cognition and emotion
  Introduction to cognitive reconstruction and behavioral training (behavioral activation, daily activity arrangement, etc)
- Case story
  Instructions and examples of cognitive reconstruction and behavioral training

**Lesson 4**

- Lesson content
  Introduction to the cognition, discussion, and reconstruction of basic beliefs
  Further introduction to behavioral training (further case conceptualization of cognition, vertical analysis of behavior, problem-solving strategies, further reconstruction of cognition and behavioral training, cognitive flash cards and cognitive motto, testing basic beliefs, etc)
- Case story
  Instructions and examples of the reconstruction of basic beliefs and behavioral training

**Lesson 5**

- Lesson content
  Further introduction to the cognition, reconstruction of basic beliefs (consolidation of positive cognition, confirming and modifying basic beliefs, etc)
  Further introduction to behavioral training (continued behavior training, etc)
  Introduction to relapse prevention and constructing relapse prevention plans
  A summary of the 5 lessons and future behavior plan, etc
- Case story
  Instructions and examples of the reconstruction of basic beliefs and behavior training
  Instructions and examples of relapse prevention and relapse prevention plans
  Instructions and examples of the summary and future plan

#### Group-Based Face-to-Face CBT

CBT participants received similar treatment modules in the same chronological order as the ICBT participants to ensure that both groups were provided with the same treatment components. There were 5 weekly sessions, each lasting 1-1.5 hours. Two experienced licensed psychologists in each LTC setting, who were blinded to the hypotheses, delivered the interventions. They were rigorously trained on the entire CBT procedure by the research clinicians before the study began to ensure that participants from different settings were treated consistently. All the group CBT sessions were conducted in a private room within the LTC setting.

#### WL Condition

Participants randomized to the WL condition were informed that they would receive an intervention within a few weeks, while no further contact or information was provided. They would choose the CBT or ICBT intervention independently after the test.

### Outcomes

The primary outcome was depressive symptoms measured by the Chinese version of the CES-D (Cronbach α=.88 in this study) [28]. Secondary measures included the Chinese version of the Geriatric Depression Scale (GDS; Cronbach α=.92 in this study) [29] and PHQ-9 (Cronbach α=.81 in this study) [24], which assess depressive symptoms. Another secondary outcome was anxiety symptoms on the Chinese version of the Generalized Anxiety Disorder 7-Item (GAD-7; Cronbach α=.83 in this study) [30]. These measurement tools have shown good reliability and validity among Chinese populations [31-34]. To assess the participants’ satisfaction with and acceptability of the ICBT, they were asked to respond with either “yes” or “no” to the following two questions: (1) “are you confident to recommend this program?” and (2) “was this program worth your time?”

### Statistical Analysis

All tests were 2-sided, and a *P* value of <.05 was considered statistically significant. Differences between the groups on baseline characteristics were compared using either 1-way ANOVA or Kruskal-Wallis tests for continuous variables and *χ*^2^ tests for categorical variables. All analyses were performed using SAS version 9.4 (SAS Institute).

Participants in the 2 intervention groups who did not start lesson 1 were excluded from the analyses. The least-squares means of outcomes were estimated based on an intention-to-treat basis, performing a mixed-effects model for repeated measures [35], which contained the time, treatment group, and time×group interaction as fixed effects. Effects were further modeled using the restricted maximum likelihood and an unstructured covariance matrix, including demographic and baseline characteristics as covariates. Mean between-group differences and mean changes for each group at each time point were analyzed using appropriate contrasts in the mixed-effects model for repeated measures.

We evaluated the effect sizes (Cohen *d*) using the criteria proposed for mixed model analysis [36-38] and calculated CIs based on the method suggested by Kelley [39]. A Cohen *d* of 0.2-0.49 was considered a small effect size, 0.5-0.79 was considered a medium effect size, and 0.8 or more was considered a large effect size [40].

We also calculated reliable change and clinically significant improvement on the CSE-D from baseline to postintervention, as well as 6- and 12-month follow-ups, according to Jacobson and Truax [41]. Reliable change was defined as a decrease of at least 3 points (using the criterion that the reliable change index must be >1.96 and using retest reliability reported for the CES-D [0.88] in the reliable change index formula) on the CES-D score, plus a CES-D postintervention score below the clinical cutoff of 16 to achieve clinically significant improvement. Next, we used a *χ*^2^ test to assess the frequency differences in reliable change and clinically significant improvements between the 3 groups. We also calculated the number needed to treat with 95% CIs to achieve 1 additional reliable change and clinically significant improvement at postintervention, relative to the WL condition [42].

## Results

### Participants’ Demographic Characteristics

Table 1 shows the demographic characteristics of the participants. Demographic characteristics and outcomes were similar for the 3 groups at baseline. Overall, the mean (SD) age was 78.60 (7.80) years. Most participants were female (215/315, 68.3%), were married (270/315, 85.7%), were graduates of junior high school or lower education (281/315, 89.2%), had low-to-medium income (298/315, 94.6%), and had no histories of MDD (257/315, 81.6%) or therapy for MDD (202/315, 64.1%). Participants were evenly recruited from public social welfare facilities (104/315, 33%), nursing homes (99/315, 31.4%), and other residential care facilities evenly (112/315, 35.6%).

**Table 1:** Demographic characteristics of the participants.

Participant characteristics		Total (N=315)	CBT^a^ (n=104)	ICBT^b^ (n=105)	WL^c^ (n=106)	*P* value	
**Sex, n (%)**							.08
	Male	100 (31.7)	30 (28.8)	42 (40)	28 (26.4)		
	Female	215 (68.3)	74 (71.2)	63 (60)	78 (73.6)		
Age (years), mean (SD)		71.6 (7.80)	71.8 (7.97)	71.2 (8.31)	71.9 (6.79)	.92	
**Study site, n (%)**							.20
	Public social welfare facilities	104 (33)	38 (36.5)	30 (28.6)	36 (34)		
	Nursing homes	99 (31.4)	33 (31.7)	30 (28.6)	36 (34)		
	Other residential care facilities	112 (35.6)	33 (31.7)	45 (42.9)	34 (32.1)		
**Marital status, n (%)**							.98
	Unmarried	8 (2.5)	3 (2.9)	2 (1.9)	3 (2.8)		
	Married	270 (85.7)	89 (85.6)	91 (86.7)	90 (84.9)		
	Divorced or widowed	37 (11.7)	12 (11.5)	12 (11.4)	13 (12.3)		
**Monthly income, n (%)**							.98
	Low	148 (47)	51 (49)	49 (46.7)	48 (45.3)		
	Medium	150 (47.6)	46 (44.2)	51 (48.6)	53 (50)		
	High	17 (5.4)	7 (6.7)	5 (4.8)	5 (4.7)		
**Education, n (%)**							.53
	Primary school or below	155 (49.2)	54 (51.9)	51 (48.6)	50 (47.2)		
	Junior high school	126 (40)	39 (37.5)	46 (43.8)	41 (38.7)		
	Senior high school	21 (6.7)	7 (6.7)	5 (4.8)	9 (8.5)		
	College or above	13 (4.1)	4 (3.8)	3 (2.9)	6 (5.7)		
**History of MDD^d^, n (%)**							.44
	No	257 (81.6)	85 (81.7)	82 (78.1)	90 (84.9)		
	Yes	58 (18.4)	19 (18.3)	23 (21.9)	16 (15.1)		
**Previous therapy for MDD, n (%)**							.53
	None	202 (64.1)	73 (70.2)	60 (57.1)	69 (65.1)		
	Antidepressant	49 (15.6)	11 (10.6)	23 (21.9)	15 (14.2)		
	Psychotherapy	44 (14)	14 (13.5)	16 (15.2)	14 (13.2)		
	Both antidepressant and psychotherapy	20 (6.3)	6 (5.8)	6 (5.7)	8 (7.5)		

^a^CBT: cognitive behavioral therapy.

^b^ICBT: internet-based cognitive behavioral therapy.

^c^WL: waiting list.

^d^MDD: major depressive disorder.

### Main Outcomes

Table 2 depicts the baseline, postintervention, 6-month follow-up, and 12-month follow-up least-squares mean and SD of outcomes for each group. The mixed model for repeated measures analyses revealed significant time×group interactions across the primary and secondary outcomes (CES-D: *F*_2,205_=3.37, *P*=.04; PHQ-9: *F*_2,220_=24.33, *P*<.001; GDS: *F*_2,199_=13.80, *P*<.001; GAD-7: *F*_2,219_=25.86, *P*<.001).

**Table 2:** Mean, SD, and effect sizes (Cohen *d*) of outcomes for study conditions.

Outcome and time point		CBT^a^, mean (SD)	ICBT^b^, mean (SD)	WL^c^, mean (SD)	Time × group, *F* test (*df*)		Significant contrasts between 2 groups		Within-group effect size (95% CI)				Between-group effect size (95% CI)		
									CBT	ICBT	WL	ICBT vs CBT		CBT vs WL	ICBT vs WL
**CES-D^d^**						3.37 (2, 205)^h^									
	Baseline	18.8 (4.15)	19.1 (4.44)	19.4 (4.37)			—^j^		—	—	—	—		—	—
	Postintervention	15.6 (3.87)	14.4 (4.26)	15.8 (3.76)			WL<ICBT^h^		0.16 (0.14 to 0.19)	0.42 (0.40 to 0.44)	0.15 (0.13 to 0.18)	0.00 (–0.02 to 0.02)		0.05 (0.02 to 0.07)	0.07 (0.04 to 0.09)
	6-month follow-up	15.8 (3.63)	14.4 (4.05)	—			—		0.17 (0.14 to 0.19)	0.43 (0.41 to 0.45)	—	0.03 (0.00-0.05)		—	—
	12-month follow-up	16.3 (3.40)	14.6 (3.71)	—			—		0.13 (0.11 to 0.16)	0.30 (0.28 to 0.33)	—	0.05 (0.01 to 0.08)		—	—
**PHQ-9^e^**						24.33 (2, 220)^i^									
	Baseline	8.0 (5.10)	8.4 (5.48)	8.1 (5.36)			—		—	—	—	—		—	—
	Postintervention	6.4 (4.74)	5.0 (2.66)	7.2 (4.62)			WL<ICBT^i^, WL<CBT^k^, CBT<ICBT^i^		0.20 (0.18 to 0.22)	0.71 (0.69 to 0.73)	0.17 (0.14 to 0.19)	0.15 (0.13 to 0.17)		0.12 (0.10 to 0.15)	0.30 (0.28 to 0.33)
	6-month follow-up	6.2 (4.46)	4.7 (2.53)	—			CBT<ICBT^i^		0.19 (0.17 to 0.21)	0.77 (0.75 to 0.80)	—	0.18 (0.16 to 0.21)		—	—
	12-month follow-up	6.5 (4.20)	5.7 (2.32)	—			CBT<ICBT^i^		0.13 (0.10 to 0.15)	0.51 (0.49 to 0.54)	—	0.15 (0.11 to 0.19)		—	—
**GDS^f^**						13.80 (2, 199)^i^									
	Baseline	16.3 (4.85)	17.2 (5.15)	16.8 (5.06)			—		—	—	—	—		—	—
	Postintervention	12.8 (4.50)	12.2 (4.94)	15.4 (4.36)			WL<ICBT^i^, WL<CBT^i^		0.06 (0.04 to 0.08)	0.18 (0.16 to 0.20)	0.04 (0.01 to 0.06)	0.05 (0.02 to 0.07)		0.06 (0.04 to 0.09)	0.10 (0.08 to 0.13)
	6-month follow-up	11.8 (4.22)	11.5 (4.70)	—			—		0.06 (0.04 to 0.08)	0.23 (0.21 to 0.25)	—	0.04 (0.02 to 0.07)		—	—
	12-month follow-up	11.5 (3.95)	12.6 (4.30)	—			—		0.06 (0.03 to 0.08)	0.17 (0.14 to 0.19)	—	0.05 (0.02 to 0.09)		—	—
**GAD-7^g^**						25.86 (2, 219)^i^									
	Baseline	7.7 (2.47)	7.9 (2.67)	7.5 (2.59)			—		—	—	—	—		—	—
	Postintervention	6.4 (2.30)	4.7 (2.56)	7.0 (2.23)			CBT<ICBT^i^, WL<ICBT^i^		0.19 (0.17 to 0.21)	0.47 (0.45 to 0.49)	0.09 (0.06 to 0.11)	0.21 (0.19 to 0.23)		0.01 (0.02 to 0.03)	0.19 (0.17 to 0.22)
	6-month follow-up	6.4 (2.16)	4.8 (2.43)	—			CBT<ICBT^i^		0.21 (0.19 to 0.23)	0.50 (0.48 to 0.52)	—	0.18 (0.15 to 0.21)		—	—
	12-month follow-up	6.7 (2.03)	4.8 (2.23)	—			CBT<ICBT^i^		0.15 (0.12 to 0.17)	0.51 (0.48 to 0.53)	—	0.18 (0.14 to 0.21)		—	—

^a^CBT: cognitive behavioral therapy.

^b^ICBT: internet-based cognitive behavioral therapy.

^c^WL: waiting list.

^d^CES-D: Center for Epidemiologic Studies Depression Scale.

^e^PHQ-9: Patient Health Questionnaire-9 Item.

^f^GDS: Geriatric Depression Scale.

^g^GAD-7: Generalized Anxiety Disorder 7-Item.

^h^*P*<.05.

^i^*P*<.001.

^j^Not available.

^k^*P*<.005.

The intervention groups showed significant improvements in almost all outcomes (*P*<.001) at postintervention and follow-up, with no significant change in the GAD-7 for the CBT group at 12-month follow-up (*P*=.07). Furthermore, the WL group showed significant improvements in the CES-D (*P*<.001) and PHQ-9 (*P*=.01). At postintervention, there were statistically significant between-group differences in the GDS and PHQ-9 scores in favor of the intervention group (all *P*<.001), and the ICBT participants had a larger reduction of the CES-D (*P*=.01) and GAD-7 scores (*P*<.001) compared with the WL participants. The ICBT participants exhibited significantly larger improvements in the PHQ-9 and GAD-7 scores than the CBT participants did over the study period (all *P*<.001). No other significant differences in the effects of ICBT and CBT were observed.

### Effect Size

#### Primary Outcome

Table 2 shows effect sizes (Cohen *d*). We observed small within-group effect sizes on the CES-D among the 3 groups at postintervention (ICBT *d*=0.42, 95% CI 0.40-0.44; CBT *d=*0.16, 95% CI 0.14-0.19; WL *d*=0.15, 95% CI 0.13-0.18), 6-month follow-up (ICBT *d*=0.43, 95% CI 0.41-0.45; CBT *d=*0.17, 95% CI 0.14-0.19), and 12-month follow-up (ICBT *d*=0.30, 95% CI 0.28-0.33; CBT *d=*0.13, 95% CI 0.11-0.16).

The between-group effect size for CES-D among the 3 groups was trivial during the study period (postintervention: ICBT vs CBT *d=*0.00, 95% CI 0.02-0.02; CBT vs WL *d*=0.05, 95% CI 0.02-0.07; ICBT vs WL *d=*0.07, 95% CI 0.04-0.09; 6-month follow-up: ICBT vs CBT *d=*0.03, 95% CI 0.00-0.05; 12-month follow-up: ICBT vs CBT *d=*0.05, 95% CI 0.01-0.08).

#### Secondary Outcome

The within-group effect sizes for secondary outcomes ranged from trivial to medium (postintervention: ICBT *d* range=0.18-0.71, CBT *d* range=0.06-0.20, WL *d* range=0.04-0.17; 6-month follow-up: ICBT *d* range=0.23-0.77, CBT *d* range=0.06-0.21; 12-month follow-up: ICBT *d* range=0.17-0.51, CBT *d* range=0.06-0.15). The between-group effect sizes for secondary outcomes were trivial (postintervention: ICBT vs CBT *d* range=0.05-0.21, ICBT vs WL *d* range=0.10-0.30, CBT vs WL *d* range=0.01-0.12; 6-month follow-up: ICBT vs CBT *d* range=0.04-0.18; 12-month follow-up: ICBT vs CBT *d* range=0.05-0.18).

### Reliable Change and Clinically Significant Improvement

The proportions of participants reporting reliable change and clinically significant improvement are shown in Table 3. We observed significantly larger rates of reliable change and clinically significant improvement in the ICBT group compared with the CBT group at postintervention (all *P*<.001) and 6-month follow-up (reliable change: *P*<.001; clinically significant improvement: *P=*.007), but these significant differences were not maintained at 12-month follow-up. The numbers needed to treat for ICBT to achieve 1 additional reliable change and clinically significant improvement were 2.89 (95% CI 2.12-4.56) and 3.16 (95% CI 2.28-5.15), respectively, and those for CBT were 6.26 (95% CI 3.45-33.84) and 5.98 (95% CI 3.48-21.01), respectively.

**Table 3:** Proportion of participants showing clinically significant improvement or reliable change based on the Center for Epidemiologic Studies Depression Scale (N=315).

Outcome and time point		CBT^a^ (n=104), n (%)	ICBT^b^ (n=105), n (%)	WL^c^ (n=106), n (%)	Chi-square (*df*)	*P* value	NNT^d^ (95% CI)	
							CBT vs WL	ICBT vs WL
**Clinically significant improvement**								
	Postintervention	38 (36.5)	54 (51.4)	21 (19.8)	22.952 (2)	<.001	5.98 (3.48-21.01)	3.16 (2.28-5.15)
	6-month follow-up	28 (26.9)	47 (44.8)	—^e^	7.227 (1)	.007	—	—
	12-month follow-up	15 (14.4)	26 (24.8)	—	3.542 (1)	.06	—	—
**Reliable change**								
	Postintervention	49 (47.1)	69 (65.7)	33 (31.1)	25.319 (2)	<.001	6.26 (3.45-33.84)	2.89 (2.12-4.56)
	6-month follow-up	41 (39.4)	66 (62.9)	—	11.484 (1)	<.001	—	—
	12-month follow-up	33 (31.7)	39 (37.1)	—	0.678 (1)	.41	—	—

^a^CBT: cognitive behavioral therapy.

^b^ICBT: internet-based cognitive behavioral therapy.

^c^WL: waiting list.

^d^NNT: number needed to treat.

^e^Not available.

### Adherence, Attrition, and Response Rates and Treatment Satisfaction

Of the ICBT and CBT participants, 79.1% (83/105) and 62.5% (65/104) completed all the lessons, respectively. On average, a mean (SD) of 4.09 (10.41) and 3.80 (7.85) lessons were completed for the ICBT and CBT groups, respectively. The mean (SD) total clinical psychologist time per participant in the ICBT group was 35.26 (29.07) minutes. In total, 88.6% (93/105), 77.1% (81/105), and 56.2% (59/105) of ICBT participants completed the postintervention, 6-month follow-up, and 12-month follow-up assessments, respectively; 83.6% (87/104), 66.4% (69/104), and 48.1% (50/104) of CBT participants completed these respective assessments. Among the participants who completed the postintervention satisfaction questionnaires, 72% of ICBT participants (67/93) and 68% of CBT participants (60/87) indicated that they would confidently recommend this program to others. Furthermore, 68% of ICBT participants (64/93) and 62% of CBT participants (54/87) found it worthwhile to participate in this program.

## Discussion

### Principal Findings

The main finding of this RCT is that clinician-guided ICBT was relatively effective in reducing depressive symptoms for older adults with sD recruited from institutional LTC settings. ICBT participants showed greater improvement in the CES-D, PHQ-9, GDS, and GAD-7 scores compared with the WL participants at postintervention. In addition, 37.10% and 24.80% of ICBT participants reported reliable change and clinically significant improvement in CES-D from pretreatment to 12-month follow-up, respectively. Furthermore, 79.05% of ICBT participants completed 5 lessons, and more than 60% reported a high degree of satisfaction.

### Comparison With Previous Work

The findings of this study are encouraging and are consistent with those of similar studies concerning ICBT for adults with sD [43]. However, both the within-group and between-group effect sizes for ICBT versus WL regarding reduction in depressive symptom severity at postintervention were lower than those in a previous trial on older adults with sD [17]. We surmise that the inferior effect might be explained by the relatively high mean age of participants in this study (71.60 years), which was substantially higher than that in the previous study (55.00 years). A recent meta-analysis showed that the therapeutic effects of ICBT for depression might diminish slightly with age due to technological challenges and limited age-appropriate components [15]. In addition, participants in this study were less educated than those in the prior study, which might have led to limited technology competency and therefore fewer ICBT benefits [44,45].

Compared with CBT participants, ICBT participants showed a significantly larger improvement in the PHQ-9 and GAD-7 at postintervention and follow-up. In addition, a significantly higher proportion of ICBT participants reported clinically significant improvement and reliable change for CES-D at postintervention and 6-month follow-up, which are consistent with the findings by Spek et al [17]. Although compelling meta-analysis evidence shows that ICBT and CBT may have equal overall benefits for depressive and anxiety symptoms [16,46], this study findings may be explained by the higher adherence among ICBT participants than among CBT participants. Hilvert-Bruce et al [47] revealed that improved adherence is an important determinant of effectiveness of both ICBT and face-to-face CBT for depression and anxiety. For other outcomes, we observed a nonsignificant trend toward improvement for ICBT compared with CBT, possibly because our program was developed based on conventional CBT. Further, ICBT participants received relatively intensive guidance, which mirrors the therapeutic alliance in face-to-face CBT, and as a result, the treatment effect of these 2 interventions might be similar. Nevertheless, most of the prior studies contrasting ICBT and CBT were based on the general population instead of older adults, and no study has reported between-group effect sizes on the outcomes of ICBT versus CBT among older adults with sD, not to mention LTC residents with sD. Thus, more research with a larger sample should be conducted to further compare ICBT with CBT for sD among LTC residents and explore adherence as a moderator of treatment effects.

To the best of our knowledge, this study is the first to demonstrate the feasibility and acceptability of ICBT for Chinese older adults with sD. Our findings are comparable to those of other trials that investigated the use of ICBT for older adults with sD or MDD in Western countries [15,17]. Chinese older adults tend to have limited technology competency and thus had difficulties in using ICBT compared with older adults from developed countries with prior experience in technology use [48]. Therefore, it is encouraging to find that the adherence, response rates, and treatment satisfaction in this study were as high as those observed in the aforementioned studies. This finding might be explained by the relatively intensive therapist-guided ICBT in this study, and given the culture of obedience to the authority of doctors among older Chinese individuals, clinician-guided ICBT may be more acceptable for elderly Chinese compared with older adults from other racial and ethnic backgrounds. In addition, ICBT participants who did not attend scheduled lessons were traced through active outreach via email or telephone. These human support strategies can decrease participant attrition rate [49] and are particularly important for older adults with limited technology competency to improve their adherence to ICBT [15].

Nevertheless, concerns remain as to whether ICBT for LTC residents with sD is as effective as that for the general older population because the effect sizes observed in this study were substantially smaller than those in previous studies on ICBT for depression among the general older population [15]. There are 4 possible reasons for these findings. First, our ICBT program was not tailored for older adults, especially those living in LTC settings who tend to be older, have lower education, and have lower levels of digital competency compared with those living at home [50]. In addition, LTC residents were more likely to have a history of MDD or a history of psychotropic medication [51], which are possible predictive factors for the lack of treatment response and slower improvement [52]. Second, our ICBT program was based on conventional CBT. However, 2 prior systematic reviews have demonstrated that although CBT for LTC residents may alleviate depression, the evidence for effectiveness was mixed [53,54]. The possible reason is that the goal of CBT is to distinguish unrealistic or exaggerated thoughts from more realistic ones [55], but LTC residents are less likely to benefit from it due to institutional policies and practices. For example, this study was conducted during the COVID-19 pandemic, a time when closed management and suspension of family visits were irregularly implemented in all LTC settings in China. This lack of physical interaction might have generated more unrealistic or exaggerated thoughts and therefore reduced the effectiveness of ICBT. Third, compared with older adults living in the community, LTC residents may feel deprived of autonomy and lack of privacy by the institutional environment [55,56], which might be detrimental to their mental health [57] and consequently reduce the effectiveness of ICBT. Fourth, our ICBT program has only 5 modules, which seem to be fewer than the usual ICBT sessions for general older populations [15]. Thus, participants in this study may have limited access to more CBT skills to alleviate depressive symptoms. In this regard, researchers should develop a tailored ICBT with additional lesson content for LTC residents, considering the unique characteristics of LTC settings, and explore its effectiveness in the future.

Notably, more than 60% of the ICBT participants failed to achieve a reliable change at 12-month follow-up; this may lower the motivation of older adults with sD for more intensive treatment, such as face-to-face CBT or pharmacotherapy [58]. Consequently, untreated LTC residents with sD may have higher risk of MDD and impaired physical and mental health [10] and may therefore become less likely to discharge to the community. Thus, future studies are needed to explore the predictive factors for nonresponders in LTC settings.

### Implications

First, our findings highlight the feasibility and potential effectiveness of ICBT for improving the mental health of older adults with sD, especially those living in LTC settings. In effect, they are likely to be discharged to the community, which may alleviate the burden on the LTC system. Considering the relatively undeveloped LTC system [22], limited resources for mental health services, and many undetected and untreated cases of sD in LTC settings in China, this study underscores the importance of developing a tailored ICBT for LTC residents with sD and promoting its use in China. Second, guided ICBT can produce equivalent overall effects as face-to-face CBT for LTC residents with sD. Nevertheless, there is a shortage of psychotherapists in Chinese LTC settings, and most of them have low professionalism [20]. In addition, because of the advantages of ICBT, including flexibility, scalability, privacy, and cost-effectiveness, ICBT can be a realistic alternative to CBT in LTC settings in China. Third, our findings provide preliminary evidence for the potential usefulness of ICBT to reduce depressive symptoms among LTC residents in China and other countries during the COVID-19 pandemic, given the closed management and visitor restrictions.

### Strengths and Limitations

The core strengths of this trial were its RCT design with direct comparison of 2 interventions, validated measures with good reliability and validity, large sample size, and a high level of adherence. The inclusion of participants with a history of MDD and on antidepressants is both a strength and a weakness; it increases the external validity of the findings but may obfuscate the effects of the intervention [52,59]. Nevertheless, several limitations should be considered. First, the choice of group CBT intervention, which has a lower degree of privacy than individual CBT, as a comparator group, may adversely affect participants’ engagement. In future studies, individual CBT may be preferable as a comparator. Second, this study included participants over 60 years old, which is a group of people in whom depression and other age-related disorders are perceived as normal in most cases. Furthermore, this study relied on self-reported measures rather than objective depression diagnoses at postintervention and follow-up. This may cause a high risk of selection bias, and results should therefore be interpreted carefully. Third, these findings may have lower generalizability because all the LTC facilities in this study were located in Zhejiang Province, one of the most economically vibrant and developed provinces in China. Thus, a further RCT should be conducted using a more diverse population from different Chinese provinces.

### Conclusions

Guided ICBT has been shown to be equally effective and acceptable as CBT for improving depressive symptoms among Chinese LTC residents with sD, and the improvements are maintained over 12 months after the intervention. Given the insufficient mental health services in LTC settings in China, ICBT has the potential to reach millions of Chinese LTC residents exposed to sD, who are at risk of being undetected and untreated.

## Appendix

Multimedia Appendix 1 CONSORT eHEALTH checklist (V.1.6.1)

## Abbreviations

CBT: cognitive behavioral therapy
CES-D: Epidemiological Studies Depression Scale
DSM-IV: Diagnostic and Statistical Manual of Mental Disorders, Fourth Edition
GAD-7: Generalized Anxiety Disorder 7-Item
GDS: Geriatric Depression Scale
ICBT: internet-based cognitive behavioral therapy
LTC: long-term care
MDD: major depressive disorder
PHQ-9: Patient Health Questionnaire–9
RCT: randomized controlled trial
SCID: Semistructured Clinical Interview for Diagnostic and Statistical Manual of Mental Disorders, Fourth Edition Axis Disorders
sD: subthreshold depression
WL: waiting list
