# Supplementary material for: Efficacy of Internet-Based Cognitive Behavioral Therapy for Subthreshold Depression Among Older Adults in Institutional Long-Term Care Settings: Pragmatic Randomized Controlled Trial
Source: J Med Internet Res. 2024 Mar 1;26:e40187. doi: 10.2196/40187 (PMC10943436; doi:10.2196/40187)
Supplement: Multimedia Appendix 1 [file jmir_v26i1e40187_app1.pdf]

CONSORT-EHEALTH (V 1.6.1) - Submission/Publication Form

The CONSORT-EHEALTH checklist is intended for authors of randomized trials evaluating web-based and Internet-based applications/interventions, including mobile interventions, electronic games (incl multiplayer games), social media, certain telehealth applications, and other interactive and/or networked electronic applications. Some of the items (e.g. all subitems under item 5 - description of the intervention) may also be applicable for other study designs.

The goal of the CONSORT EHEALTH checklist and guideline is to be
a) a guide for reporting for authors of RCTs,
b) to form a basis for appraisal of an ehealth trial (in terms of validity)

CONSORT-EHEALTH items/subitems are MANDATORY reporting items for studies published in the Journal of Medical Internet Research and other journals / scientific societies endorsing the checklist.

Items numbered 1., 2., 3., 4a., 4b etc are original CONSORT or CONSORT-NPT (non-pharmacologic treatment) items.
Items with Roman numerals (i., ii, iii, iv etc.) are CONSORT-EHEALTH extensions/clarifications.

As the CONSORT-EHEALTH checklist is still considered in a formative stage, we would ask that you also RATE ON A SCALE OF 1-5 how important/useful you feel each item is FOR THE PURPOSE OF THE CHECKLIST and reporting guideline (optional).

Mandatory reporting items are marked with a red *.
In the textboxes, either copy & paste the relevant sections from your manuscript into this form - please include any quotes from your manuscript in QUOTATION MARKS,
or answer directly by providing additional information not in the manuscript, or elaborating on why the item was not relevant for this study.

YOUR ANSWERS WILL BE PUBLISHED AS A SUPPLEMENTARY FILE TO YOUR PUBLICATION IN JMIR AND ARE CONSIDERED PART OF YOUR PUBLICATION (IF ACCEPTED).
Please fill in these questions diligently. Information will not be copyedited, so please use proper spelling and grammar, use correct capitalization, and avoid abbreviations.

DO NOT FORGET TO SAVE AS PDF _AND_ CLICK THE SUBMIT BUTTON SO YOUR ANSWERS ARE IN OUR DATABASE !!!

Citation Suggestion (if you append the pdf as Appendix we suggest to cite this paper in the caption):
Eysenbach G, CONSORT-EHEALTH Group
[illegible]

您正在编辑回复。如果您与他人共享此网址，则其他人也能编辑您的回复。 填写一份新的回复

URL: http://www.jmir.org/2011/4/e126/
doi: 10.2196/jmir.1923
PMID: 22209829

登录 Google 即可保存进度。了解详情

* 表示必填

Your name *

First Last

Yuchen Ying

Primary Affiliation (short), City, Country *

University of Toronto, Toronto, Canada

Ningbo College of Health Science, Zhejiang, Ch

Your e-mail address *

abc@gmail.com

1246401741@qq.com

Title of your manuscript *

Provide the (draft) title of your manuscript.

Efficacy of Internet-Based Cognitive Behavioral Therapy for Subthreshold Depression Among Older Adults in Institutional Long-Term Care Settings: Pragmatic Randomized Controlled Trial

您正在编辑回复。如果您与他人共享此网址，则其他人也能编辑您的回复。 填写一份新的回复

Name of your App/Software/Intervention *

If there is a short and a long/alternate name, write the short name first and add the long name in brackets.

Health psychology station

Evaluated Version (if any)

e.g. "V1", "Release 2017-03-01", "Version 2.0.27913"

您的回答

Language(s) *

What language is the intervention/app in? If multiple languages are available, separate by comma (e.g. "English, French")

English

URL of your Intervention Website or App

e.g. a direct link to the mobile app on app in appstore (itunes, Google Play), or URL of the website. If the intervention is a DVD or hardware, you can also link to an Amazon page.

您的回答

URL of an image/screenshot (optional)

您的回答

您正在编辑回复。如果您与他人共享此网址，则其他人也能编辑您的回复。 填写一份新的回复

Accessibility *

Can an enduser access the intervention presently?

- (●) access is free and open
- ( ) access only for special usergroups, not open
- ( ) access is open to everyone, but requires payment/subscription/in-app purchases
- ( ) app/intervention no longer accessible
- ( ) 其他:

Primary Medical Indication/Disease/Condition *

e.g. "Stress", "Diabetes", or define the target group in brackets after the condition, e.g. "Autism (Parents of children with)", "Alzheimers (Informal Caregivers of)"

Subthreshold Depression

Primary Outcomes measured in trial *

comma-separated list of primary outcomes reported in the trial

CES-D

Secondary/other outcomes

Are there any other outcomes the intervention is expected to affect?

您的回答

您正在编辑回复。如果您与他人共享此网址，则其他人也能编辑您的回复。 填写一份新的回复

Recommended "Dose" *

What do the instructions for users say on how often the app should be used?

- ○ Approximately Daily
- ● Approximately Weekly
- ○ Approximately Monthly
- ○ Approximately Yearly
- ○ "as needed"
- ○ 其他:

Approx. Percentage of Users (starters) still using the app as recommended after 3 months *

- ● unknown / not evaluated
- ○ 0-10%
- ○ 11-20%
- ○ 21-30%
- ○ 31-40%
- ○ 41-50%
- ○ 51-60%
- ○ 61-70%
- ○ 71%-80%
- ○ 81-90%
- ○ 91-100%
- ○ 其他:

您正在编辑回复。如果您与他人共享此网址，则其他人也能编辑您的回复。 填写一份新的回复

Overall, was the app/intervention effective? *

- (selected) yes: all primary outcomes were significantly better in intervention group vs control
- partly: SOME primary outcomes were significantly better in intervention group vs control
- no statistically significant difference between control and intervention
- potentially harmful: control was significantly better than intervention in one or more outcomes
- inconclusive: more research is needed
- 其他:

Article Preparation Status/Stage *

At which stage in your article preparation are you currently (at the time you fill in this form)

- not submitted yet - in early draft status
- not submitted yet - in late draft status, just before submission
- submitted to a journal but not reviewed yet
- submitted to a journal and after receiving initial reviewer comments
- submitted to a journal and accepted, but not published yet
- (selected) published
- 其他:

您正在编辑回复。如果您与他人共享此网址，则其他人也能编辑您的回复。 填写一份新的回复

Journal *

If you already know where you will submit this paper (or if it is already submitted), please provide the journal name (if it is not JMIR, provide the journal name under "other")

- ○ not submitted yet / unclear where I will submit this
- ◉ Journal of Medical Internet Research (JMIR)
- ○ JMIR mHealth and UHealth
- ○ JMIR Serious Games
- ○ JMIR Mental Health
- ○ JMIR Public Health
- ○ JMIR Formative Research
- ○ Other JMIR sister journal
- ○ 其他:

Is this a full powered effectiveness trial or a pilot/feasibility trial? *

- ○ Pilot/feasibility
- ◉ Fully powered

Manuscript tracking number *

If this is a JMIR submission, please provide the manuscript tracking number under "other" (The ms tracking number can be found in the submission acknowledgement email, or when you login as author in JMIR. If the paper is already published in JMIR, then the ms tracking number is the four-digit number at the end of the DOI, to be found at the bottom of each published article in JMIR)

- ○ no ms number (yet) / not (yet) submitted to / published in JMIR
- ◉ 其他: 40187

您正在编辑回复。如果您与他人共享此网址，则其他人也能编辑您的回复。 填写一份新的回复

TITLE AND ABSTRACT

1a) TITLE: Identification as a randomized trial in the title

1a) Does your paper address CONSORT item 1a? *

I.e does the title contain the phrase "Randomized Controlled Trial"? (if not, explain the reason under "other")

- (x) yes
- ( ) 其他:

1a-i) Identify the mode of delivery in the title

Identify the mode of delivery. Preferably use “web-based” and/or “mobile” and/or “electronic game” in the title. Avoid ambiguous terms like “online”, “virtual”, “interactive”. Use “Internet-based” only if Intervention includes non-web-based Internet components (e.g. email), use “computer-based” or “electronic” only if offline products are used. Use “virtual” only in the context of “virtual reality” (3-D worlds). Use “online” only in the context of “online support groups”. Complement or substitute product names with broader terms for the class of products (such as “mobile” or “smart phone” instead of “iphone”), especially if the application runs on different platforms.

	1	2	3	4	5	
subitem not at all important	( )	( )	( )	( )	( )	essential

Does your paper address subitem 1a-i? *

Copy and paste relevant sections from manuscript title (include quotes in quotation marks "like this" to indicate direct quotes from your manuscript), or elaborate on this item by providing additional information not in the ms, or briefly explain why the item is not applicable/relevant for your study

Efficacy of Internet-Based Cognitive Behavioral Therapy

您正在编辑回复。如果您与他人共享此网址，则其他人也能编辑您的回复。 填写一份新的回复

1a-ii) Non-web-based components or important co-interventions in title

Mention non-web-based components or important co-interventions in title, if any (e.g., “with telephone support”).

	1	2	3	4	5	
subitem not at all important	○	○	○	○	○	essential

Does your paper address subitem 1a-ii?

Copy and paste relevant sections from manuscript title (include quotes in quotation marks "like this" to indicate direct quotes from your manuscript), or elaborate on this item by providing additional information not in the ms, or briefly explain why the item is not applicable/relevant for your study

您的回答

1a-iii) Primary condition or target group in the title

Mention primary condition or target group in the title, if any (e.g., “for children with Type I Diabetes”) Example: A Web-based and Mobile Intervention with Telephone Support for Children with Type I Diabetes: Randomized Controlled Trial

	1	2	3	4	5	
subitem not at all important	○	○	○	○	○	essential

Does your paper address subitem 1a-iii? *

Copy and paste relevant sections from manuscript title (include quotes in quotation marks "like this" to indicate direct quotes from your manuscript), or elaborate on this item by providing additional information not in the ms, or briefly explain why the item is not applicable/relevant for your study

Subthreshold Depression Among Older Adults in Institutional Long-Term Care Settings

您正在编辑回复。如果您与他人共享此网址，则其他人也能编辑您的回复。 填写一份新的回复

1b) ABSTRACT: Structured summary of trial design, methods, results, and conclusions

NPT extension: Description of experimental treatment, comparator, care providers, centers, and blinding status.

1b-i) Key features/functionalities/components of the intervention and comparator in the METHODS section of the ABSTRACT

Mention key features/functionalities/components of the intervention and comparator in the abstract. If possible, also mention theories and principles used for designing the site. Keep in mind the needs of systematic reviewers and indexers by including important synonyms. (Note: Only report in the abstract what the main paper is reporting. If this information is missing from the main body of text, consider adding it)

	1	2	3	4	5	
subitem not at all important	○	○	○	○	○	essential

Does your paper address subitem 1b-i? *

Copy and paste relevant sections from the manuscript abstract (include quotes in quotation marks "like this" to indicate direct quotes from your manuscript), or elaborate on this item by providing additional information not in the ms, or briefly explain why the item is not applicable/relevant for your study

A total of 354 participants were randomized to ICBT, group-based CBT, or a waiting list

1b-ii) Level of human involvement in the METHODS section of the ABSTRACT

Clarify the level of human involvement in the abstract, e.g., use phrases like “fully automated” vs. “therapist/nurse/care provider/physician-assisted” (mention number and expertise of providers involved, if any). (Note: Only report in the abstract what the main paper is reporting. If this information is missing from the main body of text, consider adding it)

	1	2	3	4	5	
subitem not at all important	○	○	○	○	○	essential

您正在编辑回复。如果您与他人共享此网址，则其他人也能编辑您的回复。 填写一份新的回复

Does your paper address subitem 1b-ii?

Copy and paste relevant sections from the manuscript abstract (include quotes in quotation marks "like this" to indicate direct quotes from your manuscript), or elaborate on this item by providing additional information not in the ms, or briefly explain why the item is not applicable/relevant for your study

您的回答

1b-iii) Open vs. closed, web-based (self-assessment) vs. face-to-face assessments in the METHODS section of the ABSTRACT

Mention how participants were recruited (online vs. offline), e.g., from an open access website or from a clinic or a closed online user group (closed usergroup trial), and clarify if this was a purely web-based trial, or there were face-to-face components (as part of the intervention or for assessment). Clearly say if outcomes were self-assessed through questionnaires (as common in web-based trials). Note: In traditional offline trials, an open trial (open-label trial) is a type of clinical trial in which both the researchers and participants know which treatment is being administered. To avoid confusion, use “blinded” or “unblinded” to indicated the level of blinding instead of “open”, as “open” in web-based trials usually refers to “open access” (i.e. participants can self-enrol). (Note: Only report in the abstract what the main paper is reporting. If this information is missing from the main body of text, consider adding it)

	1	2	3	4	5	
subitem not at all important	○	○	○	○	○	essential

Does your paper address subitem 1b-iii?

Copy and paste relevant sections from the manuscript abstract (include quotes in quotation marks "like this" to indicate direct quotes from your manuscript), or elaborate on this item by providing additional information not in the ms, or briefly explain why the item is not applicable/relevant for your study

您的回答

您正在编辑回复。如果您与他人共享此网址，则其他人也能编辑您的回复。 填写一份新的回复

1b-iv) RESULTS section in abstract must contain use data

Report number of participants enrolled/assessed in each group, the use/uptake of the intervention (e.g., attrition/adherence metrics, use over time, number of logins etc.), in addition to primary/secondary outcomes. (Note: Only report in the abstract what the main paper is reporting. If this information is missing from the main body of text, consider adding it)

	1	2	3	4	5	
subitem not at all important	○	○	○	○	○	essential

Does your paper address subitem 1b-iv?

Copy and paste relevant sections from the manuscript abstract (include quotes in quotation marks "like this" to indicate direct quotes from your manuscript), or elaborate on this item by providing additional information not in the ms, or briefly explain why the item is not applicable/relevant for your study

您的回答

1b-v) CONCLUSIONS/DISCUSSION in abstract for negative trials

Conclusions/Discussions in abstract for negative trials: Discuss the primary outcome - if the trial is negative (primary outcome not changed), and the intervention was not used, discuss whether negative results are attributable to lack of uptake and discuss reasons. (Note: Only report in the abstract what the main paper is reporting. If this information is missing from the main body of text, consider adding it)

	1	2	3	4	5	
subitem not at all important	○	○	○	○	○	essential

您正在编辑回复。如果您与他人共享此网址，则其他人也能编辑您的回复。 填写一份新的回复

Does your paper address subitem 1b-v?

Copy and paste relevant sections from the manuscript abstract (include quotes in quotation marks "like this" to indicate direct quotes from your manuscript), or elaborate on this item by providing additional information not in the ms, or briefly explain why the item is not applicable/relevant for your study

您的回答

INTRODUCTION

2a) In INTRODUCTION: Scientific background and explanation of rationale

2a-i) Problem and the type of system/solution

Describe the problem and the type of system/solution that is object of the study: intended as stand-alone intervention vs. incorporated in broader health care program? Intended for a particular patient population? Goals of the intervention, e.g., being more cost-effective to other interventions, replace or complement other solutions? (Note: Details about the intervention are provided in “Methods” under 5)

	1	2	3	4	5	
subitem not at all important	○	○	○	○	○	essential

您正在编辑回复。如果您与他人共享此网址，则其他人也能编辑您的回复。 填写一份新的回复

Does your paper address subitem 2a-i? *

Copy and paste relevant sections from the manuscript (include quotes in quotation marks "like this" to indicate direct quotes from your manuscript), or elaborate on this item by providing additional information not in the ms, or briefly explain why the item is not applicable/relevant for your study

Many older Chinese adults are unlikely to seek face-to-face psychotherapy due to stigma that discourages disclosure influenced by Chinese cultural values; thus, undertreatment is common among older adults in China [18]. Of note, the overall detection rate of mild depressive symptoms among Chinese LTC residents is only 30% [19]. Considering China's 2.15 million LTC residents and the extreme shortage of its mental health workforce, especially psychological therapists in LTC settings [20], many cases of sD might go undetected and untreated. To address this issue, the Chinese government released an action plan for depression prevention and treatment to improve access to web interventions (eg, ICBT) for depression among older adults [21]. However, the efficacy and acceptability of ICBT among the older adult Chinese population, especially those in LTC settings, have not been investigated. Therefore, evidence-based research is needed to promote the use of ICBT in LTC settings in China. LTC settings in China are broadly divided into two categories: (1) institutional settings and (2) home- and community-based settings [22]. This study focused on institutional LTC facilities because they have a somewhat similar definition to that of LTC settings in previous studies [10].

2a-ii) Scientific background, rationale: What is known about the (type of) system

Scientific background, rationale: What is known about the (type of) system that is the object of the study (be sure to discuss the use of similar systems for other conditions/diagnoses, if appropiate), motivation for the study, i.e. what are the reasons for and what is the context for this specific study, from which stakeholder viewpoint is the study performed, potential impact of findings [2]. Briefly justify the choice of the comparator.

	1	2	3	4	5	
subitem not at all important	○	○	○	○	○	essential

您正在编辑回复。如果您与他人共享此网址，则其他人也能编辑您的回复。 填写一份新的回复

Does your paper address subitem 2a-ii? *

Copy and paste relevant sections from the manuscript (include quotes in quotation marks "like this" to indicate direct quotes from your manuscript), or elaborate on this item by providing additional information not in the ms, or briefly explain why the item is not applicable/relevant for your study

Subthreshold depression (sD) is a significant health burden for older adults [1]. It is even more prevalent than diagnosed clinical depression among older adults [2] and adversely affects their social functions [3], activities of daily living [4], and quality of life [5]. In addition, it is related to increased risk of major depression [6], cognitive impairment and dementia [7], physical ailments [8], and increased mortality and medical costs in older adults [9]. Furthermore, epidemiological research on older adults with sD has revealed a pattern: increasing rates of moving from community to primary care to long-term care (LTC) settings [10]. Therefore, it is necessary to develop appropriate interventions to treat sD among older adults, especially those living in LTC settings.

With respect to the treatment of depression, older adults prefer psychotherapy to pharmacotherapy [11]; cognitive behavioral therapy (CBT) has proven effective [12]. However, the treatment rate for older adults is low due to numerous barriers to face-to-face CBT, including mobility limitations, relatively high medical costs, limited number of trained therapists, and stigma [13]. Fortunately, internet-based CBT (ICBT), an intervention that can be conducted anytime and anywhere over the internet, can overcome these barriers [14]. Nevertheless, although several studies have demonstrated that ICBT may significantly reduce depressive symptoms in older adults with moderate to severe depression [15], the impact of ICBT on older adults with sD is unclear. In addition, although preliminary evidence demonstrated that guided ICBT and face-to-face CBT might be equally effective [16], only 1 prior study has demonstrated that ICBT is more effective than face-to-face CBT in treating sD among older adults living in the community [17]. However, this study did not provide a between-group effect size on the outcomes of ICBT versus CBT, and, therefore, whether ICBT is as effective as face-to-face CBT among LTC residents with sD remains unknown.

2b) In INTRODUCTION: Specific objectives or hypotheses

您正在编辑回复。如果您与他人共享此网址，则其他人也能编辑您的回复。 填写一份新的回复

Does your paper address CONSORT subitem 2b? *

Copy and paste relevant sections from the manuscript (include quotes in quotation marks "like this" to indicate direct quotes from your manuscript), or elaborate on this item by providing additional information not in the ms, or briefly explain why the item is not applicable/relevant for your study

The purpose of this study was to test the following hypotheses: (1) ICBT is more effective than a waiting list (WL) in improving depressive symptoms and other secondary outcomes, (2) ICBT is equally effective as group CBT in improving depressive symptoms and other secondary outcomes, and (3) ICBT is effective and acceptable for Chinese LTC residents.

METHODS

3a) Description of trial design (such as parallel, factorial) including allocation ratio

Does your paper address CONSORT subitem 3a? *

Copy and paste relevant sections from the manuscript (include quotes in quotation marks "like this" to indicate direct quotes from your manuscript), or elaborate on this item by providing additional information not in the ms, or briefly explain why the item is not applicable/relevant for your study

This study was a pragmatic, multicenter randomized controlled trial (RCT) with 3 arms comparing clinician-guided ICBT with group CBT and a WL control. The patients were assessed at baseline, immediately after the intervention, and at 6- and 12-month follow-ups. The interventions and WL control were not compared at 6- and 12-month follow-ups because the WL group had only 2 assessment points (ie, baseline and postintervention).

3b) Important changes to methods after trial commencement (such as eligibility criteria), with reasons

您正在编辑回复。如果您与他人共享此网址，则其他人也能编辑您的回复。 填写一份新的回复

Does your paper address CONSORT subitem 3b? *

Copy and paste relevant sections from the manuscript (include quotes in quotation marks "like this" to indicate direct quotes from your manuscript), or elaborate on this item by providing additional information not in the ms, or briefly explain why the item is not applicable/relevant for your study

There were no important changes to methods after trial commencement. Eligibility criteria remained consistent throughout.

3b-i) Bug fixes, Downtimes, Content Changes

Bug fixes, Downtimes, Content Changes: ehealth systems are often dynamic systems. A description of changes to methods therefore also includes important changes made on the intervention or comparator during the trial (e.g., major bug fixes or changes in the functionality or content) (5-iii) and other “unexpected events” that may have influenced study design such as staff changes, system failures/downtimes, etc. [2].

	1	2	3	4	5	
subitem not at all important	○	○	○	○	○	essential

Does your paper address subitem 3b-i?

Copy and paste relevant sections from the manuscript (include quotes in quotation marks "like this" to indicate direct quotes from your manuscript), or elaborate on this item by providing additional information not in the ms, or briefly explain why the item is not applicable/relevant for your study

您的回答

4a) Eligibility criteria for participants

您正在编辑回复。如果您与他人共享此网址，则其他人也能编辑您的回复。 填写一份新的回复

Does your paper address CONSORT subitem 4a? *

Copy and paste relevant sections from the manuscript (include quotes in quotation marks "like this" to indicate direct quotes from your manuscript), or elaborate on this item by providing additional information not in the ms, or briefly explain why the item is not applicable/relevant for your study

The inclusion criteria were as follows: (1) be a resident of China, (2) be 60 years or older, (3) have access to the internet, (4) have sD (defined as having a score of ≥16 on the Centre for Epidemiological Studies Depression Scale [CES-D], according to the widely used criteria in comparable studies [6,23]), (5) no suicidal ideation (indicated by a score of <2 on question 9 on the Patient Health Questionnaire–9 [PHQ-9]) [24], (6) no cognitive impairment with a score on a Mini-Mental State Examination >24 [25], (7) not currently undergoing psychotherapy, (8) no alcohol dependency, and (9) no terminal illness on clinical grounds.

The exclusion criteria were as follows: meeting the DSM-IV criteria for (1) major depressive disorder (MDD), (2) bipolar disorder, or (3) psychotic disorder.

4a-i) Computer / Internet literacy

Computer / Internet literacy is often an implicit “de facto” eligibility criterion - this should be explicitly clarified.

	1	2	3	4	5	
subitem not at all important	○	○	○	○	○	essential

Does your paper address subitem 4a-i?

Copy and paste relevant sections from the manuscript (include quotes in quotation marks "like this" to indicate direct quotes from your manuscript), or elaborate on this item by providing additional information not in the ms, or briefly explain why the item is not applicable/relevant for your study

您的回答

您正在编辑回复。如果您与他人共享此网址，则其他人也能编辑您的回复。 填写一份新的回复

4a-ii) Open vs. closed, web-based vs. face-to-face assessments:

Open vs. closed, web-based vs. face-to-face assessments: Mention how participants were recruited (online vs. offline), e.g., from an open access website or from a clinic, and clarify if this was a purely web-based trial, or there were face-to-face components (as part of the intervention or for assessment), i.e., to what degree got the study team to know the participant. In online-only trials, clarify if participants were quasi-anonymous and whether having multiple identities was possible or whether technical or logistical measures (e.g., cookies, email confirmation, phone calls) were used to detect/prevent these.

	1	2	3	4	5	
subitem not at all important	○	○	○	○	○	essential

Does your paper address subitem 4a-ii? *

Copy and paste relevant sections from the manuscript (include quotes in quotation marks "like this" to indicate direct quotes from your manuscript), or elaborate on this item by providing additional information not in the ms, or briefly explain why the item is not applicable/relevant for your study

To protect the residents' privacy, initial contact was made by the designated research liaison staff member at each LTC setting. This person asked about the residents' willingness to participate in the study, examined their health records, and identified eligible participants based on our screening criteria.

4a-iii) Information giving during recruitment

Information given during recruitment. Specify how participants were briefed for recruitment and in the informed consent procedures (e.g., publish the informed consent documentation as appendix, see also item X26), as this information may have an effect on user self-selection, user expectation and may also bias results.

	1	2	3	4	5	
subitem not at all important	○	○	○	○	○	essential

您正在编辑回复。如果您与他人共享此网址，则其他人也能编辑您的回复。 填写一份新的回复

Does your paper address subitem 4a-iii?

Copy and paste relevant sections from the manuscript (include quotes in quotation marks "like this" to indicate direct quotes from your manuscript), or elaborate on this item by providing additional information not in the ms, or briefly explain why the item is not applicable/relevant for your study

您的回答

4b) Settings and locations where the data were collected

Does your paper address CONSORT subitem 4b? *

Copy and paste relevant sections from the manuscript (include quotes in quotation marks "like this" to indicate direct quotes from your manuscript), or elaborate on this item by providing additional information not in the ms, or briefly explain why the item is not applicable/relevant for your study

A total of 618 participants were recruited via 18 LTC settings across 6 cities in Zhejiang Province. The choice of the cities and LTC settings ensured that the sample included participants from locations with different population compositions.

4b-i) Report if outcomes were (self-)assessed through online questionnaires

Clearly report if outcomes were (self-)assessed through online questionnaires (as common in web-based trials) or otherwise.

	1	2	3	4	5	
subitem not at all important	○	○	○	○	○	essential

Does your paper address subitem 4b-i? *

Copy and paste relevant sections from the manuscript (include quotes in quotation marks "like this" to indicate direct quotes from your manuscript), or elaborate on this item by providing additional information not in the ms, or briefly explain why the item is not applicable/relevant for your study

您正在编辑回复。如果您与他人共享此网址，则其他人也能编辑您的回复。 填写一份新的回复

4b-ii) Report how institutional affiliations are displayed

Report how institutional affiliations are displayed to potential participants [on ehealth media], as affiliations with prestigious hospitals or universities may affect volunteer rates, use, and reactions with regards to an intervention.(Not a required item – describe only if this may bias results)

	1	2	3	4	5	
subitem not at all important	○	○	○	○	○	essential

Does your paper address subitem 4b-ii?

Copy and paste relevant sections from the manuscript (include quotes in quotation marks "like this" to indicate direct quotes from your manuscript), or elaborate on this item by providing additional information not in the ms, or briefly explain why the item is not applicable/relevant for your study

您的回答

5) The interventions for each group with sufficient details to allow replication, including how and when they were actually administered

5-i) Mention names, credential, affiliations of the developers, sponsors, and owners

Mention names, credential, affiliations of the developers, sponsors, and owners [6] (if authors/evaluators are owners or developer of the software, this needs to be declared in a "Conflict of interest" section or mentioned elsewhere in the manuscript).

	1	2	3	4	5	
subitem not at all important	○	○	○	○	○	essential

您正在编辑回复。如果您与他人共享此网址，则其他人也能编辑您的回复。 填写一份新的回复

Does your paper address subitem 5-i?

Copy and paste relevant sections from the manuscript (include quotes in quotation marks "like this" to indicate direct quotes from your manuscript), or elaborate on this item by providing additional information not in the ms, or briefly explain why the item is not applicable/relevant for your study

您的回答

5-ii) Describe the history/development process

Describe the history/development process of the application and previous formative evaluations (e.g., focus groups, usability testing), as these will have an impact on adoption/use rates and help with interpreting results.

	1	2	3	4	5	
subitem not at all important	○	○	○	○	○	essential

Does your paper address subitem 5-ii?

Copy and paste relevant sections from the manuscript (include quotes in quotation marks "like this" to indicate direct quotes from your manuscript), or elaborate on this item by providing additional information not in the ms, or briefly explain why the item is not applicable/relevant for your study

您的回答

5-iii) Revisions and updating

Revisions and updating. Clearly mention the date and/or version number of the application/intervention (and comparator, if applicable) evaluated, or describe whether the intervention underwent major changes during the evaluation process, or whether the development and/or content was “frozen” during the trial. Describe dynamic components such as news feeds or changing content which may have an impact on the replicability of the intervention (for unexpected events see item 3b).

1	2	3	4	5
○	○	○	○	○

您正在编辑回复。如果您与他人共享此网址，则其他人也能编辑您的回复。 填写一份新的回复

Does your paper address subitem 5-iii?

Copy and paste relevant sections from the manuscript (include quotes in quotation marks "like this" to indicate direct quotes from your manuscript), or elaborate on this item by providing additional information not in the ms, or briefly explain why the item is not applicable/relevant for your study

您的回答

5-iv) Quality assurance methods

Provide information on quality assurance methods to ensure accuracy and quality of information provided [1], if applicable.

	1	2	3	4	5	
subitem not at all important	○	○	○	○	○	essential

Does your paper address subitem 5-iv?

Copy and paste relevant sections from the manuscript (include quotes in quotation marks "like this" to indicate direct quotes from your manuscript), or elaborate on this item by providing additional information not in the ms, or briefly explain why the item is not applicable/relevant for your study

您的回答

5-v) Ensure replicability by publishing the source code, and/or providing screenshots/screen-capture video, and/or providing flowcharts of the algorithms used

Ensure replicability by publishing the source code, and/or providing screenshots/screen-capture video, and/or providing flowcharts of the algorithms used. Replicability (i.e., other researchers should in principle be able to replicate the study) is a hallmark of scientific reporting.

	1	2	3	4	5	
subitem not at all important	○	○	○	○	○	essential

您正在编辑回复。如果您与他人共享此网址，则其他人也能编辑您的回复。 填写一份新的回复

Does your paper address subitem 5-v?

Copy and paste relevant sections from the manuscript (include quotes in quotation marks "like this" to indicate direct quotes from your manuscript), or elaborate on this item by providing additional information not in the ms, or briefly explain why the item is not applicable/relevant for your study

您的回答

5-vi) Digital preservation

Digital preservation: Provide the URL of the application, but as the intervention is likely to change or disappear over the course of the years; also make sure the intervention is archived (Internet Archive, webcitation.org, and/or publishing the source code or screenshots/videos alongside the article). As pages behind login screens cannot be archived, consider creating demo pages which are accessible without login.

	1	2	3	4	5	
subitem not at all important	○	○	○	○	○	essential

Does your paper address subitem 5-vi?

Copy and paste relevant sections from the manuscript (include quotes in quotation marks "like this" to indicate direct quotes from your manuscript), or elaborate on this item by providing additional information not in the ms, or briefly explain why the item is not applicable/relevant for your study

您的回答

您正在编辑回复。如果您与他人共享此网址，则其他人也能编辑您的回复。 填写一份新的回复

5-vii) Access

Access: Describe how participants accessed the application, in what setting/context, if they had to pay (or were paid) or not, whether they had to be a member of specific group. If known, describe how participants obtained “access to the platform and Internet” [1]. To ensure access for editors/reviewers/readers, consider to provide a “backdoor” login account or demo mode for reviewers/readers to explore the application (also important for archiving purposes, see vi).

	1	2	3	4	5	
subitem not at all important	○	○	○	○	○	essential

Does your paper address subitem 5-vii? *

Copy and paste relevant sections from the manuscript (include quotes in quotation marks "like this" to indicate direct quotes from your manuscript), or elaborate on this item by providing additional information not in the ms, or briefly explain why the item is not applicable/relevant for your study

Participants accessed the application through mobile phone.

5-viii) Mode of delivery, features/functionalities/components of the intervention and comparator, and the theoretical framework

Describe mode of delivery, features/functionalities/components of the intervention and comparator, and the theoretical framework [6] used to design them (instructional strategy [1], behaviour change techniques, persuasive features, etc., see e.g., [7, 8] for terminology). This includes an in-depth description of the content (including where it is coming from and who developed it) [1],” whether [and how] it is tailored to individual circumstances and allows users to track their progress and receive feedback” [6]. This also includes a description of communication delivery channels and – if computer-mediated communication is a component – whether communication was synchronous or asynchronous [6]. It also includes information on presentation strategies [1], including page design principles, average amount of text on pages, presence of hyperlinks to other resources, etc. [1].

	1	2	3	4	5	
subitem not at all important	○	○	○	○	○	essential

您正在编辑回复。如果您与他人共享此网址，则其他人也能编辑您的回复。 填写一份新的回复

Does your paper address subitem 5-viii? *

Copy and paste relevant sections from the manuscript (include quotes in quotation marks "like this" to indicate direct quotes from your manuscript), or elaborate on this item by providing additional information not in the ms, or briefly explain why the item is not applicable/relevant for your study

Internet-Based CBT
This clinician-guided ICBT is based on the principle of the Chinese version of the Handbook of Cognitive-Behavioral Therapies [26]. In this trial, the depression module was used. This module comprises 5 lessons with corresponding case stories, which were developed from real cases of Chinese patients who recovered from sD or MDD after receiving CBT, and was designed to educate the general Chinese population about practical CBT skills for depression [27]. Textbox 1 depicts each lesson's descriptions. Although the duration of lessons could vary among participants, they were advised to undertake at least 1 lesson per week in an individual format. The role of clinicians, who were blinded to the hypotheses, was to provide technical assistance and encouragement to participants during the study.

Group-Based Face-to-Face CBT
CBT participants received similar treatment modules in the same chronological order as the ICBT participants to ensure that both groups were provided with the same treatment components. There were 5 weekly sessions, each lasting 1-1.5 hours. Two experienced licensed psychologists in each LTC setting, who were blinded to the hypotheses, delivered the interventions. They were rigorously trained on the entire CBT procedure by the research clinicians before the study began to ensure that participants from different settings were treated consistently. All the group CBT sessions were conducted in a private room within the LTC setting.

Waiting List
Participants randomized to the WL condition were informed that they would receive an intervention within a few weeks, while no further contact or information was provided. They would choose the CBT or ICBT intervention independently after the test.

5-ix) Describe use parameters

Describe use parameters (e.g., intended "doses" and optimal timing for use). Clarify what instructions or recommendations were given to the user, e.g., regarding timing, frequency, heaviness of use, if any, or was the intervention used ad libitum.

	1	2	3	4	5	
subitem not at all important	○	○	○	○	○	essential

您正在编辑回复。如果您与他人共享此网址，则其他人也能编辑您的回复。 填写一份新的回复

Does your paper address subitem 5-ix?

Copy and paste relevant sections from the manuscript (include quotes in quotation marks "like this" to indicate direct quotes from your manuscript), or elaborate on this item by providing additional information not in the ms, or briefly explain why the item is not applicable/relevant for your study

您的回答

5-x) Clarify the level of human involvement

Clarify the level of human involvement (care providers or health professionals, also technical assistance) in the e-intervention or as co-intervention (detail number and expertise of professionals involved, if any, as well as “type of assistance offered, the timing and frequency of the support, how it is initiated, and the medium by which the assistance is delivered”. It may be necessary to distinguish between the level of human involvement required for the trial, and the level of human involvement required for a routine application outside of a RCT setting (discuss under item 21 – generalizability).

	1	2	3	4	5	
subitem not at all important	○	○	○	○	○	essential

Does your paper address subitem 5-x?

Copy and paste relevant sections from the manuscript (include quotes in quotation marks "like this" to indicate direct quotes from your manuscript), or elaborate on this item by providing additional information not in the ms, or briefly explain why the item is not applicable/relevant for your study

您的回答

您正在编辑回复。如果您与他人共享此网址，则其他人也能编辑您的回复。 填写一份新的回复

5-xi) Report any prompts/reminders used

Report any prompts/reminders used: Clarify if there were prompts (letters, emails, phone calls, SMS) to use the application, what triggered them, frequency etc. It may be necessary to distinguish between the level of prompts/reminders required for the trial, and the level of prompts/reminders for a routine application outside of a RCT setting (discuss under item 21 – generalizability).

	1	2	3	4	5	
subitem not at all important	○	○	○	○	○	essential

Does your paper address subitem 5-xi? *

Copy and paste relevant sections from the manuscript (include quotes in quotation marks "like this" to indicate direct quotes from your manuscript), or elaborate on this item by providing additional information not in the ms, or briefly explain why the item is not applicable/relevant for your study

The role of clinicians, who were blinded to the hypotheses, was to provide technical assistance and encouragement to participants during the study.

5-xii) Describe any co-interventions (incl. training/support)

Describe any co-interventions (incl. training/support): Clearly state any interventions that are provided in addition to the targeted eHealth intervention, as ehealth intervention may not be designed as stand-alone intervention. This includes training sessions and support [1]. It may be necessary to distinguish between the level of training required for the trial, and the level of training for a routine application outside of a RCT setting (discuss under item 21 – generalizability.

	1	2	3	4	5	
subitem not at all important	○	○	○	○	○	essential

您正在编辑回复。如果您与他人共享此网址，则其他人也能编辑您的回复。 填写一份新的回复

Does your paper address subitem 5-xii? *

Copy and paste relevant sections from the manuscript (include quotes in quotation marks "like this" to indicate direct quotes from your manuscript), or elaborate on this item by providing additional information not in the ms, or briefly explain why the item is not applicable/relevant for your study

There were no co-interventions.

6a) Completely defined pre-specified primary and secondary outcome measures, including how and when they were assessed

Does your paper address CONSORT subitem 6a? *

Copy and paste relevant sections from the manuscript (include quotes in quotation marks "like this" to indicate direct quotes from your manuscript), or elaborate on this item by providing additional information not in the ms, or briefly explain why the item is not applicable/relevant for your study

The primary outcome was depressive symptoms measured by the Chinese version of the CES-D (Cronbach α=.88 in this study) [28]. Secondary measures included the Chinese version of the Geriatric Depression Scale (GDS; Cronbach α=.92 in this study) [29] and PHQ-9 (Cronbach α=.81 in this study) [24], which assess depressive symptoms. Another secondary outcome was anxiety symptoms on the Chinese version of the Generalized Anxiety Disorder 7-Item (GAD-7; Cronbach α=.83 in this study) [30]. These measurement tools have shown good reliability and validity among Chinese populations [31-34].

6a-i) Online questionnaires: describe if they were validated for online use and apply CHERRIES items to describe how the questionnaires were designed/deployed

If outcomes were obtained through online questionnaires, describe if they were validated for online use and apply CHERRIES items to describe how the questionnaires were designed/deployed [9].

	1	2	3	4	5	
subitem not at all important	○	○	○	○	○	essential

您正在编辑回复。如果您与他人共享此网址，则其他人也能编辑您的回复。 填写一份新的回复

Does your paper address subitem 6a-i?

Copy and paste relevant sections from manuscript text

您的回答

6a-ii) Describe whether and how “use” (including intensity of use/dosage) was defined/measured/monitored

Describe whether and how “use” (including intensity of use/dosage) was defined/measured/monitored (logins, logfile analysis, etc.). Use/adoption metrics are important process outcomes that should be reported in any ehealth trial.

	1	2	3	4	5	
subitem not at all important	○	○	○	○	○	essential

Does your paper address subitem 6a-ii?

Copy and paste relevant sections from manuscript text

您的回答

6a-iii) Describe whether, how, and when qualitative feedback from participants was obtained

Describe whether, how, and when qualitative feedback from participants was obtained (e.g., through emails, feedback forms, interviews, focus groups).

	1	2	3	4	5	
subitem not at all important	○	○	○	○	○	essential

您正在编辑回复。如果您与他人共享此网址，则其他人也能编辑您的回复。 填写一份新的回复

Does your paper address subitem 6a-iii?

Copy and paste relevant sections from manuscript text

您的回答

6b) Any changes to trial outcomes after the trial commenced, with reasons

Does your paper address CONSORT subitem 6b? *

Copy and paste relevant sections from the manuscript (include quotes in quotation marks "like this" to indicate direct quotes from your manuscript), or elaborate on this item by providing additional information not in the ms, or briefly explain why the item is not applicable/relevant for your study

There were no changes to trial outcomes after the trial commenced.

7a) How sample size was determined

NPT: When applicable, details of whether and how the clustering by care provides or centers was addressed

7a-i) Describe whether and how expected attrition was taken into account when calculating the sample size

Describe whether and how expected attrition was taken into account when calculating the sample size.

	1	2	3	4	5	
subitem not at all important	○	○	○	○	○	essential

您正在编辑回复。如果您与他人共享此网址，则其他人也能编辑您的回复。 填写一份新的回复

Does your paper address subitem 7a-i?

Copy and paste relevant sections from manuscript title (include quotes in quotation marks "like this" to indicate direct quotes from your manuscript), or elaborate on this item by providing additional information not in the ms, or briefly explain why the item is not applicable/relevant for your study

您的回答

7b) When applicable, explanation of any interim analyses and stopping guidelines

Does your paper address CONSORT subitem 7b? *

Copy and paste relevant sections from the manuscript (include quotes in quotation marks "like this" to indicate direct quotes from your manuscript), or elaborate on this item by providing additional information not in the ms, or briefly explain why the item is not applicable/relevant for your study

There were no interim analyses and stopping guidelines.

8a) Method used to generate the random allocation sequence

NPT: When applicable, how care providers were allocated to each trial group

Does your paper address CONSORT subitem 8a? *

Copy and paste relevant sections from the manuscript (include quotes in quotation marks "like this" to indicate direct quotes from your manuscript), or elaborate on this item by providing additional information not in the ms, or briefly explain why the item is not applicable/relevant for your study

Randomization was performed at a ratio of 1:1:1 in blocks of 6 using an web randomizer by an external researcher from another institution

8b) Type of randomisation; details of any restriction (such as blocking and block size)

您正在编辑回复。如果您与他人共享此网址，则其他人也能编辑您的回复。 填写一份新的回复

Does your paper address CONSORT subitem 8b? *

Copy and paste relevant sections from the manuscript (include quotes in quotation marks "like this" to indicate direct quotes from your manuscript), or elaborate on this item by providing additional information not in the ms, or briefly explain why the item is not applicable/relevant for your study

Randomization was performed at a ratio of 1:1:1 in blocks of 6 using an web randomizer by an external researcher from another institution

9) Mechanism used to implement the random allocation sequence (such as sequentially numbered containers), describing any steps taken to conceal the sequence until interventions were assigned

Does your paper address CONSORT subitem 9? *

Copy and paste relevant sections from the manuscript (include quotes in quotation marks "like this" to indicate direct quotes from your manuscript), or elaborate on this item by providing additional information not in the ms, or briefly explain why the item is not applicable/relevant for your study

Each allocation was kept in sequentially numbered, sealed opaque envelopes.

10) Who generated the random allocation sequence, who enrolled participants, and who assigned participants to interventions

Does your paper address CONSORT subitem 10? *

Copy and paste relevant sections from the manuscript (include quotes in quotation marks "like this" to indicate direct quotes from your manuscript), or elaborate on this item by providing additional information not in the ms, or briefly explain why the item is not applicable/relevant for your study

Randomization was performed at a ratio of 1:1:1 in blocks of 6 using an web randomizer by an external researcher from another institution

您正在编辑回复。如果您与他人共享此网址，则其他人也能编辑您的回复。 填写一份新的回复

11a) If done, who was blinded after assignment to interventions (for example, participants, care providers, those assessing outcomes) and how

NPT: Whether or not administering co-interventions were blinded to group assignment

11a-i) Specify who was blinded, and who wasn't

Specify who was blinded, and who wasn't. Usually, in web-based trials it is not possible to blind the participants [1, 3] (this should be clearly acknowledged), but it may be possible to blind outcome assessors, those doing data analysis or those administering co-interventions (if any).

	1	2	3	4	5	
subitem not at all important	○	○	○	○	○	essential

Does your paper address subitem 11a-i? *

Copy and paste relevant sections from the manuscript (include quotes in quotation marks "like this" to indicate direct quotes from your manuscript), or elaborate on this item by providing additional information not in the ms, or briefly explain why the item is not applicable/relevant for your study

Clinical psychologists conducting SCID were blinded to the group assignments. Except for the WL group as a control, none of the participants were blinded to group allocation.

11a-ii) Discuss e.g., whether participants knew which intervention was the "intervention of interest" and which one was the "comparator"

Informed consent procedures (4a-ii) can create biases and certain expectations - discuss e.g., whether participants knew which intervention was the "intervention of interest" and which one was the "comparator".

	1	2	3	4	5	
subitem not at all important	○	○	○	○	○	essential

您正在编辑回复。如果您与他人共享此网址，则其他人也能编辑您的回复。 填写一份新的回复

Does your paper address subitem 11a-ii?

Copy and paste relevant sections from the manuscript (include quotes in quotation marks "like this" to indicate direct quotes from your manuscript), or elaborate on this item by providing additional information not in the ms, or briefly explain why the item is not applicable/relevant for your study

您的回答

11b) If relevant, description of the similarity of interventions

(this item is usually not relevant for ehealth trials as it refers to similarity of a placebo or sham intervention to a active medication/intervention)

Does your paper address CONSORT subitem 11b? *

Copy and paste relevant sections from the manuscript (include quotes in quotation marks "like this" to indicate direct quotes from your manuscript), or elaborate on this item by providing additional information not in the ms, or briefly explain why the item is not applicable/relevant for your study

CBT participants received similar treatment modules in the same chronological order as the ICBT participants to ensure that both groups were provided with the same treatment components.

12a) Statistical methods used to compare groups for primary and secondary outcomes

NPT: When applicable, details of whether and how the clustering by care providers or centers was addressed

您正在编辑回复。如果您与他人共享此网址，则其他人也能编辑您的回复。 填写一份新的回复

Does your paper address CONSORT subitem 12a? *

Copy and paste relevant sections from the manuscript (include quotes in quotation marks "like this" to indicate direct quotes from your manuscript), or elaborate on this item by providing additional information not in the ms, or briefly explain why the item is not applicable/relevant for your study

The least-squares means of outcomes were estimated based on an intention-to-treat basis, performing a mixed-effects model for repeated measures [35], which contained the time, treatment group, and time×group interaction as fixed effects. Effects were further modeled using the restricted maximum likelihood and an unstructured covariance matrix, including demographic and baseline characteristics as covariates. Mean between-group differences and mean changes for each group at each time point were analyzed using appropriate contrasts in the mixed-effects model for repeated measures.

12a-i) Imputation techniques to deal with attrition / missing values

Imputation techniques to deal with attrition / missing values: Not all participants will use the intervention/comparator as intended and attrition is typically high in ehealth trials. Specify how participants who did not use the application or dropped out from the trial were treated in the statistical analysis (a complete case analysis is strongly discouraged, and simple imputation techniques such as LOCF may also be problematic [4]).

	1	2	3	4	5	
subitem not at all important	○	○	○	○	○	essential

Does your paper address subitem 12a-i? *

Copy and paste relevant sections from the manuscript (include quotes in quotation marks "like this" to indicate direct quotes from your manuscript), or elaborate on this item by providing additional information not in the ms, or briefly explain why the item is not applicable/relevant for your study

Participants in the 2 intervention groups who did not start lesson 1 were excluded from the analyses.

12b) Methods for additional analyses, such as subgroup analyses and adjusted analyses

您正在编辑回复。如果您与他人共享此网址，则其他人也能编辑您的回复。 填写一份新的回复

Does your paper address CONSORT subitem 12b? *

Copy and paste relevant sections from the manuscript (include quotes in quotation marks "like this" to indicate direct quotes from your manuscript), or elaborate on this item by providing additional information not in the ms, or briefly explain why the item is not applicable/relevant for your study

We also calculated reliable change and clinically significant improvement on the CSE-D from baseline to postintervention, as well as 6- and 12-month follow-up, according to Jacobson and Truax [41]. Reliable change was defined as a decrease of at least 3 points (using the criterion that the reliable change index must be >1.96 and using retest reliability reported for the CES-D [0.88] in the reliable change index formula) on the CES-D score, plus a CES-D postintervention score below the clinical cutoff of 16 to achieve clinically significant improvement. Next, we used a χ2 test to assess the frequency differences in reliable change and clinically significant improvements between the 3 groups. We also calculated the number needed to treat with 95% CIs to achieve 1 additional reliable change and clinically significant improvement at postintervention, relative to the WL condition [42].

X26) REB/IRB Approval and Ethical Considerations [recommended as subheading under "Methods"] (not a CONSORT item)

X26-i) Comment on ethics committee approval

	1	2	3	4	5	
subitem not at all important	○	○	○	○	○	essential

Does your paper address subitem X26-i?

Copy and paste relevant sections from the manuscript (include quotes in quotation marks "like this" to indicate direct quotes from your manuscript), or elaborate on this item by providing additional information not in the ms, or briefly explain why the item is not applicable/relevant for your study

您的回答

您正在编辑回复。如果您与他人共享此网址，则其他人也能编辑您的回复。 填写一份新的回复

x26-ii) Outline informed consent procedures

Outline informed consent procedures e.g., if consent was obtained offline or online (how? Checkbox, etc.?), and what information was provided (see 4a-ii). See [6] for some items to be included in informed consent documents.

	1	2	3	4	5	
subitem not at all important	○	○	○	○	○	essential

Does your paper address subitem X26-ii?

Copy and paste relevant sections from the manuscript (include quotes in quotation marks "like this" to indicate direct quotes from your manuscript), or elaborate on this item by providing additional information not in the ms, or briefly explain why the item is not applicable/relevant for your study

您的回答

X26-iii) Safety and security procedures

Safety and security procedures, incl. privacy considerations, and any steps taken to reduce the likelihood or detection of harm (e.g., education and training, availability of a hotline)

	1	2	3	4	5	
subitem not at all important	○	○	○	○	○	essential

Does your paper address subitem X26-iii?

Copy and paste relevant sections from the manuscript (include quotes in quotation marks "like this" to indicate direct quotes from your manuscript), or elaborate on this item by providing additional information not in the ms, or briefly explain why the item is not applicable/relevant for your study

您的回答

您正在编辑回复。如果您与他人共享此网址，则其他人也能编辑您的回复。 填写一份新的回复

13a) For each group, the numbers of participants who were randomly assigned, received intended treatment, and were analysed for the primary outcome

NPT: The number of care providers or centers performing the intervention in each group and the number of patients treated by each care provider in each center

Does your paper address CONSORT subitem 13a? *

Copy and paste relevant sections from the manuscript (include quotes in quotation marks "like this" to indicate direct quotes from your manuscript), or elaborate on this item by providing additional information not in the ms, or briefly explain why the item is not applicable/relevant for your study

and then randomized to ICBT (n=118), CBT (n=119), or WL (n=117).

13b) For each group, losses and exclusions after randomisation, together with reasons

Does your paper address CONSORT subitem 13b? (NOTE: Preferably, this is shown in a CONSORT flow diagram) *

Copy and paste relevant sections from the manuscript (include quotes in quotation marks "like this" to indicate direct quotes from your manuscript), or elaborate on this item by providing additional information not in the ms, or briefly explain why the item is not applicable/relevant for your study

There were no losses and exclusions after randomisation.

13b-i) Attrition diagram

Strongly recommended: An attrition diagram (e.g., proportion of participants still logging in or using the intervention/comparator in each group plotted over time, similar to a survival curve) or other figures or tables demonstrating usage/dose/engagement.

	1	2	3	4	5	
subitem not at all important	○	○	○	○	○	essential

您正在编辑回复。如果您与他人共享此网址，则其他人也能编辑您的回复。 填写一份新的回复

Does your paper address subitem 13b-i?

Copy and paste relevant sections from the manuscript or cite the figure number if applicable (include quotes in quotation marks "like this" to indicate direct quotes from your manuscript), or elaborate on this item by providing additional information not in the ms, or briefly explain why the item is not applicable/relevant for your study

您的回答

14a) Dates defining the periods of recruitment and follow-up

Does your paper address CONSORT subitem 14a? *

Copy and paste relevant sections from the manuscript (include quotes in quotation marks "like this" to indicate direct quotes from your manuscript), or elaborate on this item by providing additional information not in the ms, or briefly explain why the item is not applicable/relevant for your study

Patient participants were recruited between April 14, 2020 and October 29， 2020.

Follow-up was conducted on postintervention day and 12-month later.

14a-i) Indicate if critical “secular events” fell into the study period

Indicate if critical “secular events” fell into the study period, e.g., significant changes in Internet resources available or “changes in computer hardware or Internet delivery resources”

	1	2	3	4	5	
subitem not at all important	○	○	○	○	○	essential

您正在编辑回复。如果您与他人共享此网址，则其他人也能编辑您的回复。 填写一份新的回复

Does your paper address subitem 14a-i?

Copy and paste relevant sections from the manuscript (include quotes in quotation marks "like this" to indicate direct quotes from your manuscript), or elaborate on this item by providing additional information not in the ms, or briefly explain why the item is not applicable/relevant for your study

您的回答

14b) Why the trial ended or was stopped (early)

Does your paper address CONSORT subitem 14b? *

Copy and paste relevant sections from the manuscript (include quotes in quotation marks "like this" to indicate direct quotes from your manuscript), or elaborate on this item by providing additional information not in the ms, or briefly explain why the item is not applicable/relevant for your study

The trial ended when we achieved the enrollment numbers needed to power the study

15) A table showing baseline demographic and clinical characteristics for each group

NPT: When applicable, a description of care providers (case volume, qualification, expertise, etc.) and centers (volume) in each group

您正在编辑回复。如果您与他人共享此网址，则其他人也能编辑您的回复。 填写一份新的回复

Does your paper address CONSORT subitem 15? *

Copy and paste relevant sections from the manuscript (include quotes in quotation marks "like this" to indicate direct quotes from your manuscript), or elaborate on this item by providing additional information not in the ms, or briefly explain why the item is not applicable/relevant for your study

Participant characteristics	Total (N=315)	CBTa (n=104)	ICBTb (n=105)	WLc (n=106)	P value
Gender, n (%)					.08
Male	100 (31.7)	30 (28.8)	42 (40.0)	28 (26.4)	
Female	215 (68.3)	74 (71.2)	63 (60.0)	78 (73.6)	
Age, mean (SD)	71.6 (7.80)	71.8 (7.97)	71.2 (8.31)	71.9 (6.79)	.92
Study site, n (%)					.20
Public social welfare facilities	104 (33.0)	38 (36.5)	30 (28.6)	36 (34.0)	
Nursing homes	99 (31.4)	33 (31.7)	30 (28.6)	36 (34.0)	
Other residential care facilities	112 (35.6)	33 (31.7)	45 (42.9)	34 (32.1)	
Marital status, n (%)					.98
Unmarried	8 (2.5)	3 (2.9)	2 (1.9)	3 (2.8)	
Married	270 (85.7)	89 (85.6)	91 (86.7)	90 (84.9)	
Divorced or widowed	37 (11.7)	12 (11.5)	12 (11.4)	13 (12.3)	
Monthly income, n (%)					.98
Low	148 (47.0)	51 (49.0)	49 (46.7)	48 (45.3)	
Medium	150 (47.6)	46 (44.2)	51 (48.6)	53 (50.0)	
High	17 (5.4)	7 (6.7)	5 (4.8)	5 (4.7)	
Education, n (%)					.53
Primary school or below	155 (49.2)	54 (51.9)	51 (48.6)	50 (47.2)	
Junior high school	126 (40.0)	39 (37.5)	46 (43.8)	41 (38.7)	
Senior high school	21 (6.7)	7 (6.7)	5 (4.8)	9 (8.5)	
College or above	13 (4.1)	4 (3.8)	3 (2.9)	6 (5.7)	
History of MDDd, n (%)					.44
No	257 (81.6)	85 (81.7)	82 (78.1)	90 (84.9)	
Yes	58 (18.4)	19 (18.3)	23 (21.9)	16 (15.1)	
Previous therapy for MDD, n (%)					.53
None	202 (64.1)	73 (70.2)	60 (57.1)	69 (65.1)	
Antidepressant	49 (15.6)	11 (10.6)	23 (21.9)	15 (14.2)	
Psychotherapy	44 (14.0)	14 (13.5)	16 (15.2)	14 (13.2)	
Both antidepressant and psychotherapy	20 (6.3)	6 (5.8)	6 (5.7)	8 (7.5)	

您正在编辑回复。如果您与他人共享此网址，则其他人也能编辑您的回复。 填写一份新的回复

15-i) Report demographics associated with digital divide issues

In ehealth trials it is particularly important to report demographics associated with digital divide issues, such as age, education, gender, social-economic status, computer/Internet/ehealth literacy of the participants, if known.

	1	2	3	4	5	
subitem not at all important	O	O	O	O	O	essential

Does your paper address subitem 15-i? *

Copy and paste relevant sections from the manuscript (include quotes in quotation marks "like this" to indicate direct quotes from your manuscript), or elaborate on this item by providing additional information not in the ms, or briefly explain why the item is not applicable/relevant for your study

Participant characteristics	Total (N=315)	CBTa (n=104)	ICBTb (n=105)	WLc (n=106)	P value
Gender, n (%)					.08
Male	100 (31.7)	30 (28.8)	42 (40.0)	28 (26.4)	
Female	215 (68.3)	74 (71.2)	63 (60.0)	78 (73.6)	
Age, mean (SD)	71.6 (7.80)	71.8 (7.97)	71.2 (8.31)	71.9 (6.79)	.92
Monthly income, n (%)					.98
Low	148 (47.0)	51 (49.0)	49 (46.7)	48 (45.3)	
Medium	150 (47.6)	46 (44.2)	51 (48.6)	53 (50.0)	
High	17 (5.4)	7 (6.7)	5 (4.8)	5 (4.7)	
Education, n (%)					.53
Primary school or below	155 (49.2)	54 (51.9)	51 (48.6)	50 (47.2)	
Junior high school	126 (40.0)	39 (37.5)	46 (43.8)	41 (38.7)	
Senior high school	21 (6.7)	7 (6.7)	5 (4.8)	9 (8.5)	
College or above	13 (4.1)	4 (3.8)	3 (2.9)	6 (5.7)	

16) For each group, number of participants (denominator) included in each analysis and whether the analysis was by original assigned groups

您正在编辑回复。如果您与他人共享此网址，则其他人也能编辑您的回复。 填写一份新的回复

16-i) Report multiple “denominators” and provide definitions

Report multiple “denominators” and provide definitions: Report N’s (and effect sizes) “across a range of study participation [and use] thresholds” [1], e.g., N exposed, N consented, N used more than x times, N used more than y weeks, N participants “used” the intervention/comparator at specific pre-defined time points of interest (in absolute and relative numbers per group). Always clearly define “use” of the intervention.

1 2 3 4 5

subitem not at all important ○ ○ ○ ○ ○ essential

Does your paper address subitem 16-i? *

Copy and paste relevant sections from the manuscript (include quotes in quotation marks "like this" to indicate direct quotes from your manuscript), or elaborate on this item by providing additional information not in the ms, or briefly explain why the item is not applicable/relevant for your study

Outcome CBTa (n=104), n (%) ICBTb (n=105), n (%) WLc (n=106), n (%) Chi-square (df) P NNTd (95% CI)
CBT vs WL ICBT vs WL

Clinically significant improvement
Postintervention 38 (36.5) 54 (51.4) 21 (19.8) 22.952 (2) <.001 5.98 (3.48-21.01) 3.16 (2.28-5.15)
6-month follow-up 28 (26.9) 47 (44.8) —e 7.227 (1) .007 — —
12-month follow-up 15 (14.4) 26 (24.8) — 3.542 (1) .06 — —
Reliable change
Postintervention 49 (47.1) 69 (65.7) 33 (31.1) 25.319 (2) <.001 6.26 (3.45-33.84) 2.89 (2.12-4.56)
6-month follow-up 41 (39.4) 66 (62.9) — 11.484 (1) <.001 — —
12-month follow-up 33 (31.7) 39 (37.1) — 0.678 (1) .41 — —

16-ii) Primary analysis should be intent-to-treat

Primary analysis should be intent-to-treat, secondary analyses could include comparing only “users”, with the appropriate caveats that this is no longer a randomized sample (see 18-i).

1 2 3 4 5

您正在编辑回复。如果您与他人共享此网址，则其他人也能编辑您的回复。 填写一份新的回复

Does your paper address subitem 16-ii?

Copy and paste relevant sections from the manuscript (include quotes in quotation marks "like this" to indicate direct quotes from your manuscript), or elaborate on this item by providing additional information not in the ms, or briefly explain why the item is not applicable/relevant for your study

您的回答

17a) For each primary and secondary outcome, results for each group, and the estimated effect size and its precision (such as 95% confidence interval)

您正在编辑回复。如果您与他人共享此网址，则其他人也能编辑您的回复。 填写一份新的回复

Does your paper address CONSORT subitem 17a? *

Copy and paste relevant sections from the manuscript (include quotes in quotation marks "like this" to indicate direct quotes from your manuscript), or elaborate on this item by providing additional information not in the ms, or briefly explain why the item is not applicable/relevant for your study

Outcome CBTa, mean (SD) ICBTb, mean (SD) WLc, mean (SD) Significant contrasts between 2 groups Within-group effect size (95% CI) Between-group effect size (95% CI)
CBT ICBT WL ICBT vs CBT CBT vs WL ICBT vs WL

CES-Dd,e
Baseline 18.8 (4.15) 19.1 (4.44) 19.4 (4.37) —f — — — — — —
Postintervention 15.6 (3.87) 14.4 (4.26) 15.8 (3.76) WL<ICBTg 0.16 (0.14-0.19) 0.42 (0.40-0.44) 0.15 (0.13-0.18) 0.00 (-0.02-0.02) 0.05 (0.02-0.07) 0.07 (0.04-0.09)
6-month follow-up 15.8 (3.63) 14.4 (4.05) — — 0.17 (0.14-0.19) 0.43 (0.41-0.45) — 0.03 (0.00-0.05) — —
12-month follow-up 16.3 (3.40) 14.6 (3.71) — — 0.13 (0.11-0.16) 0.30 (0.28-0.33) — 0.05 (0.01-0.08) — —
PHQ-9h,i
Baseline 8.0 (5.10) 8.4 (5.48) 8.1 (5.36) — — — — — — —
Postintervention 6.4 (4.74) 5.0 (2.66) 7.2 (4.62) WL<ICBTj, WL<CBTk, CBT<ICBTj 0.20 (0.18-0.22) 0.71 (0.69-0.73) 0.17 (0.14-0.19) 0.15 (0.13-0.17) 0.12 (0.10-0.15) 0.30 (0.28-0.33)
6-month follow-up 6.2 (4.46) 4.7 (2.53) — CBT<ICBTj 0.19 (0.17-0.21) 0.77 (0.75-0.80) — 0.18 (0.16-0.21) — —
12-month follow-up 6.5 (4.20) 5.7 (2.32) — CBT<ICBTj 0.13 (0.10-0.15) 0.51 (0.49-0.54) — 0.15 (0.11-0.19) — —
GDSl,m
Baseline 16.3 (4.85) 17.2 (5.15) 16.8 (5.06) — — — — — — —
Postintervention 12.8 (4.50) 12.2 (4.94) 15.4 (4.36) WL<ICBTj, WL<CBTj 0.06 (0.04-0.08) 0.18 (0.16-0.20) 0.04 (0.01-0.06) 0.05 (0.02-0.07) 0.06 (0.04-0.09) 0.10 (0.08-0.13)
6-month follow-up 11.8 (4.22) 11.5 (4.70) — — 0.06 (0.04-0.08) 0.23 (0.21-0.25) — 0.04 (0.02-0.07) — —
12-month follow-up 11.5 (3.95) 12.6 (4.30) — — 0.06 (0.03-0.08) 0.17 (0.14-0.19) — 0.05 (0.02-0.09) — —
GAD-7n,o
Baseline 7.7 (2.47) 7.9 (2.67) 7.5 (2.59) — — — — — — —
Postintervention 6.4 (2.30) 4.7 (2.56) 7.0 (2.23) CBT<ICBTj, WL<ICBTj 0.19 (0.17-0.21) 0.47 (0.45-0.49) 0.09 (0.06-0.11) 0.21 (0.19-0.23) 0.01 (0.02-0.03) 0.19 (0.17-0.22)
6-month follow-up 6.4 (2.16) 4.8 (2.43) — CBT<ICBTj 0.21 (0.19-0.23) 0.50 (0.48-0.52) — 0.18 (0.15-0.21) — —
12-month follow-up 6.7 (2.03) 4.8 (2.23) — CBT<ICBTj 0.15 (0.12-0.17) 0.51 (0.48-0.53) — 0.18 (0.14-0.21) — —

您正在编辑回复。如果您与他人共享此网址，则其他人也能编辑您的回复。 填写一份新的回复

17a-i) Presentation of process outcomes such as metrics of use and intensity of use

In addition to primary/secondary (clinical) outcomes, the presentation of process outcomes such as metrics of use and intensity of use (dose, exposure) and their operational definitions is critical. This does not only refer to metrics of attrition (13-b) (often a binary variable), but also to more continuous exposure metrics such as "average session length". These must be accompanied by a technical description how a metric like a "session" is defined (e.g., timeout after idle time) [1] (report under item 6a).

	1	2	3	4	5	
subitem not at all important	○	○	○	○	○	essential

Does your paper address subitem 17a-i?

Copy and paste relevant sections from the manuscript (include quotes in quotation marks "like this" to indicate direct quotes from your manuscript), or elaborate on this item by providing additional information not in the ms, or briefly explain why the item is not applicable/relevant for your study

您的回答

17b) For binary outcomes, presentation of both absolute and relative effect sizes is recommended

您正在编辑回复。如果您与他人共享此网址，则其他人也能编辑您的回复。 填写一份新的回复

Does your paper address CONSORT subitem 17b? *

Copy and paste relevant sections from the manuscript (include quotes in quotation marks "like this" to indicate direct quotes from your manuscript), or elaborate on this item by providing additional information not in the ms, or briefly explain why the item is not applicable/relevant for your study

Outcome CBTa (n=104), n (%) ICBTb (n=105), n (%) WLc (n=106), n (%) Chi-square (df) P NNTd (95% CI)

CBT vs WL ICBT vs WL

Clinically significant improvement

Postintervention 38 (36.5) 54 (51.4) 21 (19.8) 22.952 (2) <.001 5.98 (3.48-21.01) 3.16 (2.28-5.15)

6-month follow-up 28 (26.9) 47 (44.8) —e 7.227 (1) .007 — —

12-month follow-up 15 (14.4) 26 (24.8) — 3.542 (1) .06 — —

Reliable change

Postintervention 49 (47.1) 69 (65.7) 33 (31.1) 25.319 (2) <.001 6.26 (3.45-33.84) 2.89 (2.12-4.56)

6-month follow-up 41 (39.4) 66 (62.9) — 11.484 (1) <.001 — —

12-month follow-up 33 (31.7) 39 (37.1) — 0.678 (1) .41 — —

18) Results of any other analyses performed, including subgroup analyses and adjusted analyses, distinguishing pre-specified from exploratory

Does your paper address CONSORT subitem 18? *

Copy and paste relevant sections from the manuscript (include quotes in quotation marks "like this" to indicate direct quotes from your manuscript), or elaborate on this item by providing additional information not in the ms, or briefly explain why the item is not applicable/relevant for your study

There were no other analyses performed.

您正在编辑回复。如果您与他人共享此网址，则其他人也能编辑您的回复。 填写一份新的回复

18-i) Subgroup analysis of comparing only users

A subgroup analysis of comparing only users is not uncommon in ehealth trials, but if done, it must be stressed that this is a self-selected sample and no longer an unbiased sample from a randomized trial (see 16-iii).

	1	2	3	4	5	
subitem not at all important	○	○	○	○	○	essential

Does your paper address subitem 18-i?

Copy and paste relevant sections from the manuscript (include quotes in quotation marks "like this" to indicate direct quotes from your manuscript), or elaborate on this item by providing additional information not in the ms, or briefly explain why the item is not applicable/relevant for your study

您的回答

19) All important harms or unintended effects in each group

(for specific guidance see CONSORT for harms)

Does your paper address CONSORT subitem 19? *

Copy and paste relevant sections from the manuscript (include quotes in quotation marks "like this" to indicate direct quotes from your manuscript), or elaborate on this item by providing additional information not in the ms, or briefly explain why the item is not applicable/relevant for your study

There were no important harms or unintended effects in each group.

您正在编辑回复。如果您与他人共享此网址，则其他人也能编辑您的回复。 填写一份新的回复

19-i) Include privacy breaches, technical problems

Include privacy breaches, technical problems. This does not only include physical “harm” to participants, but also incidents such as perceived or real privacy breaches [1], technical problems, and other unexpected/unintended incidents. “Unintended effects” also includes unintended positive effects [2].

	1	2	3	4	5	
subitem not at all important	○	○	○	○	○	essential

Does your paper address subitem 19-i?

Copy and paste relevant sections from the manuscript (include quotes in quotation marks "like this" to indicate direct quotes from your manuscript), or elaborate on this item by providing additional information not in the ms, or briefly explain why the item is not applicable/relevant for your study

您的回答

19-ii) Include qualitative feedback from participants or observations from staff/researchers

Include qualitative feedback from participants or observations from staff/researchers, if available, on strengths and shortcomings of the application, especially if they point to unintended/unexpected effects or uses. This includes (if available) reasons for why people did or did not use the application as intended by the developers.

	1	2	3	4	5	
subitem not at all important	○	○	○	○	○	essential

Does your paper address subitem 19-ii?

Copy and paste relevant sections from the manuscript (include quotes in quotation marks "like this" to indicate direct quotes from your manuscript), or elaborate on this item by providing additional information not in the ms, or briefly explain why the item is not applicable/relevant for your study

您正在编辑回复。如果您与他人共享此网址，则其他人也能编辑您的回复。 填写一份新的回复

DISCUSSION

22) Interpretation consistent with results, balancing benefits and harms, and considering other relevant evidence

NPT: In addition, take into account the choice of the comparator, lack of or partial blinding, and unequal expertise of care providers or centers in each group

22-i) Restate study questions and summarize the answers suggested by the data, starting with primary outcomes and process outcomes (use)

Restate study questions and summarize the answers suggested by the data, starting with primary outcomes and process outcomes (use).

	1	2	3	4	5	
subitem not at all important	○	○	○	○	○	essential

Does your paper address subitem 22-i? *

Copy and paste relevant sections from the manuscript (include quotes in quotation marks "like this" to indicate direct quotes from your manuscript), or elaborate on this item by providing additional information not in the ms, or briefly explain why the item is not applicable/relevant for your study

The main finding of this RCT is that clinician-guided ICBT was relatively effective in reducing depressive symptoms for older adults with sD recruited from institutional LTC settings. ICBT participants showed greater improvement in the CES-D, PHQ-9, GDS, and GAD-7 scores compared with the WL participants at postintervention. In addition, 37.10% and 24.80% of ICBT participants reported reliable change and clinically significant improvement in CES-D from pretreatment to 12-month follow-up, respectively. Furthermore, 79.05% of ICBT participants completed 5 lessons, and more than 60% reported a high degree of satisfaction.

您正在编辑回复。如果您与他人共享此网址，则其他人也能编辑您的回复。 填写一份新的回复

22-ii) Highlight unanswered new questions, suggest future research

Highlight unanswered new questions, suggest future research.

	1	2	3	4	5	
subitem not at all important	○	○	○	○	○	essential

Does your paper address subitem 22-ii?

Copy and paste relevant sections from the manuscript (include quotes in quotation marks "like this" to indicate direct quotes from your manuscript), or elaborate on this item by providing additional information not in the ms, or briefly explain why the item is not applicable/relevant for your study

您的回答

20) Trial limitations, addressing sources of potential bias, imprecision, and, if relevant, multiplicity of analyses

20-i) Typical limitations in ehealth trials

Typical limitations in ehealth trials: Participants in ehealth trials are rarely blinded. Ehealth trials often look at a multiplicity of outcomes, increasing risk for a Type I error. Discuss biases due to non-use of the intervention/usability issues, biases through informed consent procedures, unexpected events.

	1	2	3	4	5	
subitem not at all important	○	○	○	○	○	essential

您正在编辑回复。如果您与他人共享此网址，则其他人也能编辑您的回复。 填写一份新的回复

Does your paper address subitem 20-i? *

Copy and paste relevant sections from the manuscript (include quotes in quotation marks "like this" to indicate direct quotes from your manuscript), or elaborate on this item by providing additional information not in the ms, or briefly explain why the item is not applicable/relevant for your study

Furthermore, this study relied on self-reported measures rather than objective depression diagnoses at postintervention and follow-up. This may cause a high risk of selection bias, and results should therefore be interpreted carefully.

21) Generalisability (external validity, applicability) of the trial findings

NPT: External validity of the trial findings according to the intervention, comparators, patients, and care providers or centers involved in the trial

21-i) Generalizability to other populations

Generalizability to other populations: In particular, discuss generalizability to a general Internet population, outside of a RCT setting, and general patient population, including applicability of the study results for other organizations

	1	2	3	4	5	
subitem not at all important	○	○	○	○	○	essential

Does your paper address subitem 21-i?

Copy and paste relevant sections from the manuscript (include quotes in quotation marks "like this" to indicate direct quotes from your manuscript), or elaborate on this item by providing additional information not in the ms, or briefly explain why the item is not applicable/relevant for your study

您的回答

您正在编辑回复。如果您与他人共享此网址，则其他人也能编辑您的回复。 填写一份新的回复

21-ii) Discuss if there were elements in the RCT that would be different in a routine application setting

Discuss if there were elements in the RCT that would be different in a routine application setting (e.g., prompts/reminders, more human involvement, training sessions or other co-interventions) and what impact the omission of these elements could have on use, adoption, or outcomes if the intervention is applied outside of a RCT setting.

	1	2	3	4	5	
subitem not at all important	○	○	○	○	○	essential

Does your paper address subitem 21-ii?

Copy and paste relevant sections from the manuscript (include quotes in quotation marks "like this" to indicate direct quotes from your manuscript), or elaborate on this item by providing additional information not in the ms, or briefly explain why the item is not applicable/relevant for your study

您的回答

OTHER INFORMATION

23) Registration number and name of trial registry

Does your paper address CONSORT subitem 23? *

Copy and paste relevant sections from the manuscript (include quotes in quotation marks "like this" to indicate direct quotes from your manuscript), or elaborate on this item by providing additional information not in the ms, or briefly explain why the item is not applicable/relevant for your study

Chinese Clinical Trial Registry ChiCTR2000030697.

24) Where the full trial protocol can be accessed, if available

您正在编辑回复。如果您与他人共享此网址，则其他人也能编辑您的回复。 填写一份新的回复

Does your paper address CONSORT subitem 24? *

Cite a Multimedia Appendix, other reference, or copy and paste relevant sections from the manuscript (include quotes in quotation marks "like this" to indicate direct quotes from your manuscript), or elaborate on this item by providing additional information not in the ms, or briefly explain why the item is not applicable/relevant for your study

The full trial protocol can not be accessed.

25) Sources of funding and other support (such as supply of drugs), role of funders

Does your paper address CONSORT subitem 25? *

Copy and paste relevant sections from the manuscript (include quotes in quotation marks "like this" to indicate direct quotes from your manuscript), or elaborate on this item by providing additional information not in the ms, or briefly explain why the item is not applicable/relevant for your study

The study was approved the Medical and Health Science and Technology Plan Project of Zhejiang Province (grant 2021KY335), Ningbo Philosophy and Social Planning Project (grant G21-3-ZX94), Zhejiang Province Public Welfare Technology Application Research Project (grant LGF21H090004), General Scientific Research Project of Department of Education of Zhejiang Province (grant Y201942547), Science and Technology Innovation Activity Plan of Zhejiang University Student & XinMiao Talents Program (grants 2021R464003 and 2020R479003).

X27) Conflicts of Interest (not a CONSORT item)

X27-i) State the relation of the study team towards the system being evaluated

In addition to the usual declaration of interests (financial or otherwise), also state the relation of the study team towards the system being evaluated, i.e., state if the authors/evaluators are distinct from or identical with the developers/sponsors of the intervention.

	1	2	3	4	5	
subitem not at all important	○	○	○	○	○	essential

您正在编辑回复。如果您与他人共享此网址，则其他人也能编辑您的回复。 填写一份新的回复

Does your paper address subitem X27-i?

Copy and paste relevant sections from the manuscript (include quotes in quotation marks "like this" to indicate direct quotes from your manuscript), or elaborate on this item by providing additional information not in the ms, or briefly explain why the item is not applicable/relevant for your study

您的回答

About the CONSORT EHEALTH checklist

As a result of using this checklist, did you make changes in your manuscript? *

- ○ yes, major changes
- ○ yes, minor changes
- ◉ no

What were the most important changes you made as a result of using this checklist?

您的回答

How much time did you spend on going through the checklist INCLUDING making changes in your manuscript *

I spend 2 hours on going through the checklist.

您正在编辑回复。如果您与他人共享此网址，则其他人也能编辑您的回复。 填写一份新的回复

As a result of using this checklist, do you think your manuscript has improved? *

- (●) yes
- ( ) no
- ( ) 其他:

Would you like to become involved in the CONSORT EHEALTH group?

This would involve for example becoming involved in participating in a workshop and writing an "Explanation and Elaboration" document

- ( ) yes
- ( ) no
- ( ) 其他:

Any other comments or questions on CONSORT EHEALTH

您的回答

STOP - Save this form as PDF before you click submit

To generate a record that you filled in this form, we recommend to generate a PDF of this page (on a Mac, simply select "print" and then select "print as PDF") before you submit it.

When you submit your (revised) paper to JMIR, please upload the PDF as supplementary file.

Don't worry if some text in the textboxes is cut off, as we still have the complete information in our database. Thank you!

Final step: Click submit !

Click submit so we have your answers in our database!

您正在编辑回复。如果您与他人共享此网址，则其他人也能编辑您的回复。 填写一份新的回复

切勿通过 Google 表单提交密码。

此内容不是由 Google 所创建，Google 不对其作任何担保。 举报滥用行为 - 服务条款 - 隐私权政策

Google 表单

您正在编辑回复。如果您与他人共享此网址，则其他人也能编辑您的回复。 填写一份新的回复

您正在编辑回复。如果您与他人共享此网址，则其他人也能编辑您的回复。 填写一份新的回复
